# NLRP10 maintains epidermal homeostasis by promoting keratinocyte survival and P63-dependent differentiation and barrier function

**DOI:** 10.1038/s41419-024-07146-y

**Published:** 2024-10-18

**Authors:** Yeonhee Cho, Zhongzheng Cao, Xin Luo, Jennifer J. Tian, Renee R. Hukkanen, Rajaa Hussien, Belinda Cancilla, Priyanka Chowdhury, Fei Li, Shining Ma, Edward L. LaGory, Mark Schroeder, Amanda Dusenberry, Leslie Marshall, Jenn Hawkins, Menno van Lookeren Campagne, Yi Zhou

**Affiliations:** 1 Inflammation Research, Amgen Inc., South San Francisco, CA USA; 2 Amgen R&D Postdoctoral Fellows Program, South San Francisco, CA USA; 3 Center for Research Acceleration by Digital Innovation, Amgen Inc., South San Francisco, CA USA; 4 Translational Safety & Bioanalytical Sciences, Amgen Inc., South San Francisco, CA USA; 5 Translational Safety & Bioanalytical Sciences, Amgen Inc, Cambridge, MA USA; 6 Structural Biology, Amgen Inc., South San Francisco, CA USA; 7 Pharmacokinetics and Drug Metabolism, Amgen Inc., South San Francisco, CA USA; 8 Clinical Biomarkers, Amgen Inc, Thousand Oaks, CA USA

**Keywords:** Apoptosis, Mechanisms of disease, Atopic dermatitis, Differentiation, Lipidomics

## Abstract

Atopic dermatitis (AD) is a common chronic inflammatory skin disorder characterized by disrupted epidermal barrier function and aberrant immune responses. Despite recent developments in new therapeutics for AD, there is still a large unmet medical need for disease management due to the complex and multifactorial nature of AD. Recent genome-wide association studies (GWAS) have identified NLRP10 as a susceptible gene for AD but the physiological role of NLRP10 in skin homeostasis and AD remains unknown. Here we show that NLRP10 is downregulated in AD skin samples. Using an air-lift human skin equivalent culture, we demonstrate that NLRP10 promotes keratinocyte survival and is required for epidermal differentiation and barrier function. Mechanistically, NLRP10 limits cell death by preventing the recruitment of caspase-8 to the death inducing signaling complex (DISC) and by inhibiting its subsequent activation. NLRP10 also stabilizes p63, the master regulator of keratinocyte differentiation, to drive proper keratinocyte differentiation and to reinforce the barrier function. Our findings underscore NLRP10 as a key player in atopic dermatitis pathogenesis, highlighting NLRP10 as a potential target for therapeutic intervention to restore skin barrier function and homeostasis in AD.

## Introduction

Atopic dermatitis (AD), characterized by intense itch and recurrent eczematous lesions, is a common inflammatory skin disease [1]. The intricate interplay between genetic factors and environmental triggers underscores the complexity of AD pathophysiology [1, 2]. Disruption of epidermal barrier function caused by genetic defects or environmental insults is suggested to be the primary event in development of AD [3]. Topical corticosteroids and emollients, phototherapy and targeted small molecule inhibitors and biologics have been prescribed to manage inflammation in AD [1, 2, 4, 5]. However, disease phenotype heterogeneity based on age, ethnicity, disease duration, genetic variations and molecular endotypes advocates for a patient-targeted precision medicine approach for better disease treatment and prevention [5, 6].

Recent large-scale genome-wide association studies (GWAS) have identified several variants near the NLRP10 locus that associate with AD [7–11]. An AD risk intergenic single-nucleotide polymorphism (SNP) rs878860 has been suggested to reside within an enhancer region that interacts with NLRP10 by targeted chromosome conformation capture analysis [12], and the AD risk variant was found to down-regulate NLRP10 expression [13]. Another missense variant rs59039403 located in the NLRP10 coding region was identified to associate with reduced risk of AD in the Japanese population [7–10]. These genetic studies validated NLRP10 as a susceptible gene for AD.

NLRP10 belongs to the NACHT-, Leucine rich Repeat (LRR)- and Pyrin domain (PYD)-containing Proteins (NLRPs) family. NLRPs play critical roles in the innate immune response following recognition of specific pathogen- or damage-associated molecular patterns by promoting the assembly and activation of inflammasome and other signaling complexes. Unlike other NLRPs, NLRP10 is the only NLRP family protein that lacks the putative ligand binding LRR domain and was initially proposed as a negative regulator of the inflammasome pathway [14, 15]. However, existing literature has reported controversial data suggesting NLRP10 might either promote or suppress inflammasome activation [16–19]. Studies using *Nlrp10*-deficient mice or human cell lines suggested either a protective role against various pathogen infections [16, 20–22] or a pathogenic and pro-inflammatory role [23–25] of NLRP10 depending on the species and nature of the stimulus. NLRP10 was reported to express in the skin, tongue, testis, heart, spleen and colon in mice [16, 18, 21–24], whereas its expression in humans appeared to be more restricted to skin [17, 24]. It was also reported that the homotypic interaction of human NLRP10 PYD domain is distinct from that of the mouse homolog by structural analysis [26], adding another layer of complexity when interpreting human NLRP10 function from data generated in mouse studies. Despite the specific expression pattern of NLRP10 in the human skin and the genetic association with AD, the physiological role of NLRP10 in regulating epidermis homeostasis and its potential significance in the pathogenesis of AD remain underexplored.

In this study, we show that NLRP10 expression is reduced in the epidermis of AD patients. Using *NLRP10*-deficient primary human keratinocytes and three-dimensional human skin equivalent cultures, we demonstrate that NLRP10 regulates keratinocyte homeostasis and barrier function by limiting keratinocyte cell death and promoting p63 expression and signaling. These data highlight the integral role of NLRP10 in maintaining normal epidermal function and provide insights into the development of novel therapies for atopic dermatitis by targeting NLRP10.

## Results

### NLRP10 expression is downregulated in AD skin

**Fig. 1:**
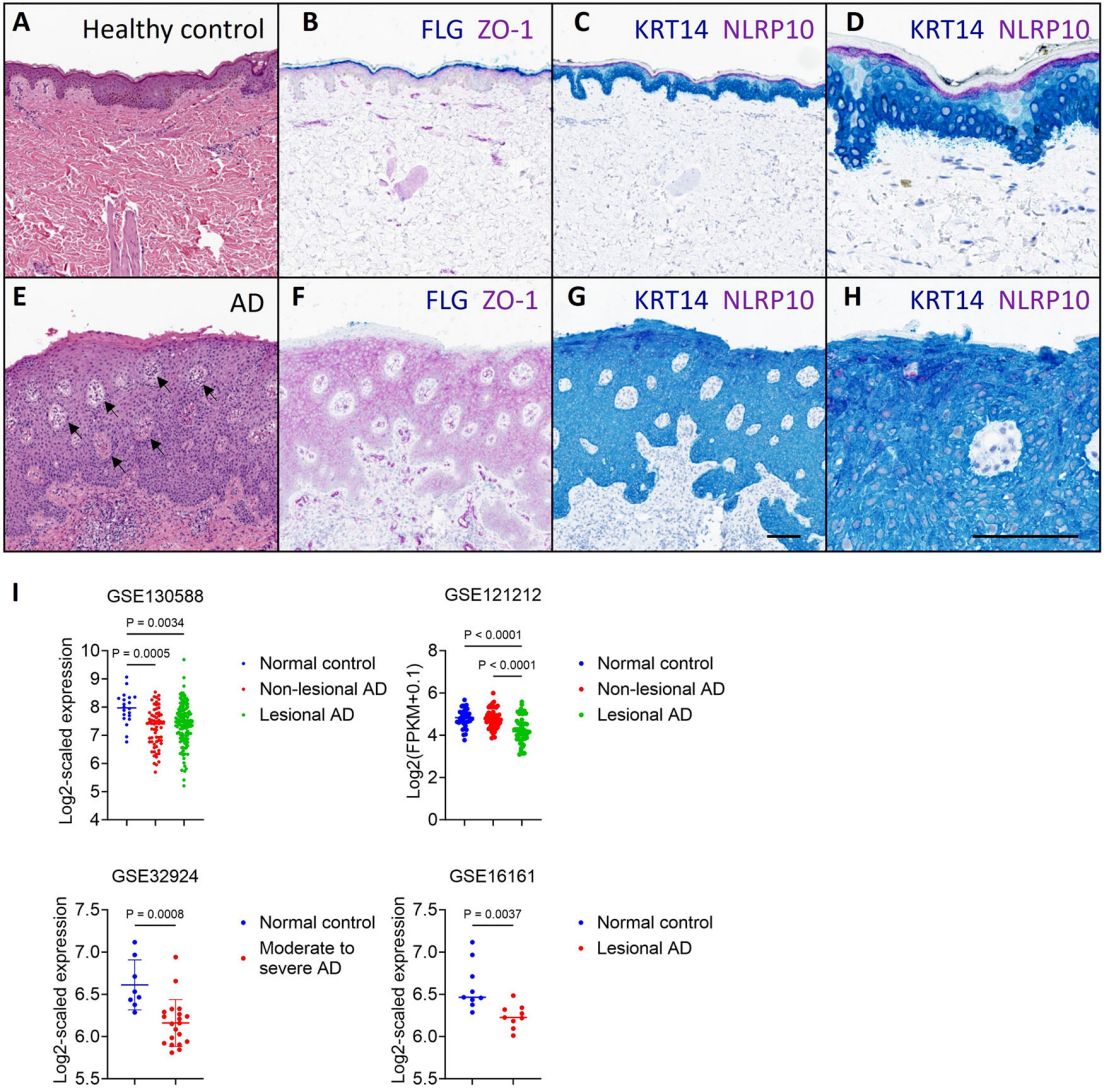
NLRP10 is downregulated in AD. Representative images of healthy skin (**A**–**D**) and severe atopic dermatitis (**E**–**H**). Samples stained with H&E (**A**, **E**): Compared to healthy skin (**A**), AD skin (**E**) demonstrates epidermal thickening (acanthosis) and multiple foci of epidermal edema (spongiosis) as exemplified by arrows. Samples stained with ZO-1 and Filaggrin (**B**, **F**): In normal skin (**B**), ZO-1 is expressed at tight junctions of the stratum granulosum (and upper stratum spinosum) and Filaggrin is expressed in terminally differentiated keratinocytes (cytoplasmic expression within the stratum corneum and lucidum). In AD (**F**), ZO-1 expression extends throughout the stratum spinosum, diffusely within the cytoplasm (not limited to cell junctions). Filaggrin expression is lost with severe AD. Samples stained with NLRP10 and Cytokeratin14 (**C**, **D** and **G**, **H**): In control skin (**C**, **D**), NLRP10 is cytoplasmic within the stratum granulosum. With severe AD (**G**, **H**), its staining is reduced. **D** and **H** Are higher magnification images of (**C**) and (**G**) respectively. Scale bar (**A**–**C**, **E**–**G**): 100 µm; scale bar (**D**, **H**): 50 µm. **I** Gene expression profile of NLRP10 in normal and AD samples from four different study cohorts showing reduced NLRP10 expression in AD. Data represent mean $\pm$ S.D. and statistical significance was determined using one-way ANOVA for GSE130588 and GSE121212 or two tailed student *t*-test for GSE32924 and GSE16161. GSE130588 (*n* = 20 for normal control, *n* = 64 for non-lesional AD; *n* = 124 for lesional AD); GSE121212 (*n* = 37 for normal control, *n* = 54 for non-lesional AD; *n* = 55 for lesional AD); GSE32924 (*n* = 8 for normal control, *n* = 20 for moderate to severe AD); GSE16161 (*n* = 9 for normal control, *n* = 9 for lesional AD).

We first analyzed the expression of NLRP10 in humans using the Genotype-Tissue Expression (GTEx) dataset (Supplementary Fig. 1A). Consistent with previous reports [17, 24], *NLRP10* transcript was specifically expressed in the human skin tissues and cultured fibroblasts. We validated the expression of NLRP10 in primary normal human epidermal keratinocytes (NHEKs) and normal human dermal fibroblasts (NHDFs) but could not detect the expression in human peripheral blood mononuclear cells (hPBMCs) (Supplementary Fig. 1B). To further visualize the expression pattern of NLRP10, we developed an immunohistochemistry (IHC) assay to detect NLRP10 in NLRP10-overexpressing CHO cells and human skin sections (Supplementary Fig. 1C, D). We then examined biopsy samples from healthy controls and AD patients. In control skin biopsies, filaggrin, a key protein involved in skin barrier function, was detected in terminally differentiated keratinocytes (Fig. 1A, B). NLRP10 was highly expressed within the stratum granulosum layer, and the expression was largely undetectable in the stratum spinosum and basal layers (Fig. 1C, D and Supplementary Fig. 1D), in accordance with a previous report showing strong upregulation of NLRP10 during keratinocyte differentiation [27]. In contrast, AD skin biopsies displayed typical pathological features of acanthosis and spongiosis (Fig. 1E). A reduction in expression of filaggrin was observed in AD (Fig. 1F). NLRP10 expression in the stratum granulosum was discontinuous and the overall expression level was reduced in AD (Fig. 1G, H). To further corroborate our findings, we queried publicly available transcriptome datasets generated from healthy and AD skin samples from four different cohorts [28–31]. Consistently, *NLRP10* transcript levels were significantly decreased in AD (Fig. 1I). We also analyzed transcriptome studies in various skin diseases that showed differential expression of NLRP10 between lesional skin samples and healthy control samples using the DiseaseLand dataset (Qiagen Omicsoft) (Supplementary Fig. 1E). NLRP10 showed a consistent reduction of expression in AD, whereas its expression was more variable in other skin diseases. Together, these data demonstrate a reduction of NLRP10 expression in AD and implicate a potential role of NLRP10 in AD pathophysiology.

### NLRP10 promotes epidermal differentiation and barrier function

**Fig. 2:**
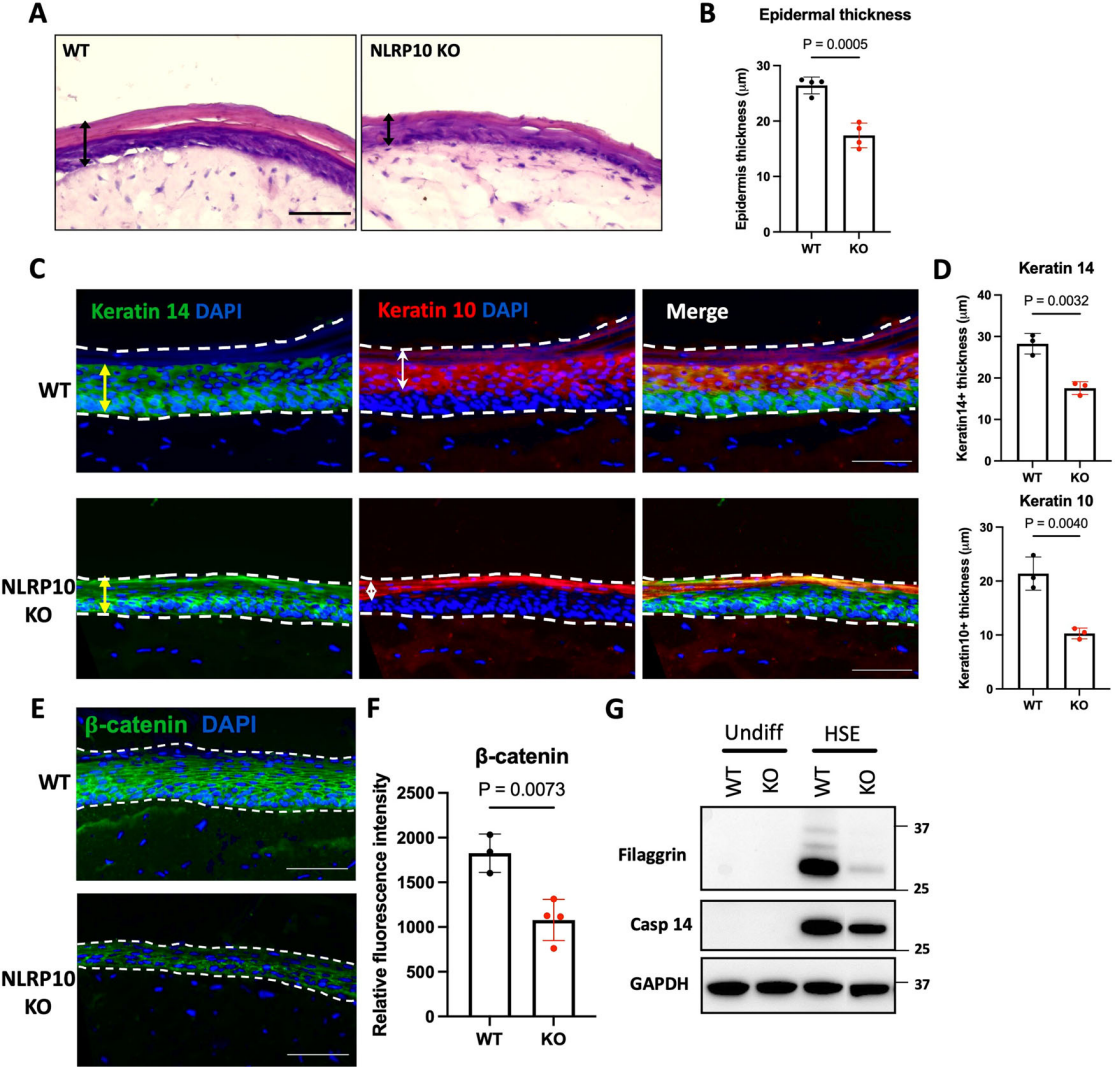
NLRP10 promotes epidermal differentiation. **A**, **B** Representative images of H&E staining (**A**) and quantification of epidermal thickness (**B**) of human skin equivalent (HSE) developed from WT or NLRP10 KO NHEKs (*n* = 4). **C**, **D** Representative images (**C**) and quantification of thickness (**D**) of Keratin 10+ and Keratin 14+ layers in HSE (*n* = 3). Yellow arrow lines highlight Keratin 14+ layers and white arrow lines indicates Keratin 10+ layers. **E**, **F** Representative images (**E**) and quantification of relative fluorescence intensity (**F**) of β-catenin in WT and NLRP10 KO HSEs (*n* = 3 for WT, *n* = 4 for KO). **G** Filaggrin and caspase-14 (Casp-14) from WT and NLRP10 KO undifferentiated NHEKs and HSEs were analyzed by Western blotting. Data represent mean $\pm$ S.D. and statistical significance was determined using two tailed student *t*-test. Dashed lines highlight the epidermis region within the HSEs. Scale bar: 20 µm.

Proper differentiation of keratinocytes into stratum corneum is critical for maintaining skin homeostasis and barrier function. Epidermal barrier dysfunction is a key contributing factor for AD [32]. The elevated expression of NLRP10 in differentiated keratinocytes prompted us to investigate whether NLRP10 plays a role in epidermal differentiation. To test this hypothesis, we evaluated if expression of *NLRP10* in the human skin GTEx dataset correlates with the level of the epidermal differentiation complex (EDC) genes, which encode major proteins involved in epidermal differentiation [33]. *NLRP10* expression was associated with the expression of multiple components of the EDC family genes, including *FLG2*, *FLG*, *LOR*, *LCE2D*, *LCE1C*, *SPRR2E*, *SPRR2G*, *CRNN* and *RPTN* (Supplementary Fig. 2). To directly assess if NLRP10 engages in epidermal differentiation, we utilized a human skin equivalent (HSE) culture system in which NHEKs differentiate into stratified epidermal layers resembling native human skin with the support of collagen-embedded dermal fibroblasts in an air-lift culture condition (Supplementary Fig. 3A). In the HSEs, keratin 14 (KRT14) labeled basal and proliferative keratinocytes, whereas keratin 10 (KRT10) was a marker of more matured keratinocytes (Supplementary Fig. 3A). To interrogate the role of NLRP10 in skin homeostasis, we generated NLRP10 knockout (KO) primary NHEKs using CRISPR/Cas9 technology. HSEs derived from NLRP10 KO NHEKs displayed significant reduction of overall epidermal thickness compared to HSEs derived from WT NHEKs (Fig. 2A, B). Ki67 staining in the HSEs showed comparable numbers of Ki67+ cells within the basal layers, ruling out the possibility that reduced epidermal thickness was caused by inadequate cell proliferation (Supplementary Fig. 3B). Immunofluorescent staining revealed a prominent decrease in the thickness of both KRT14+ basal and proliferative layers and KRT10+ differentiated layers (Fig. 2C, D). Adherens junction protein β-catenin was abundant at the cell-cell junctions in WT HSEs, whereas junctional β-catenin level was markedly diminished in NLRP10 KO HSEs (Fig. 2E, F). Furthermore, two important proteins involved in keratinocyte terminal differentiation, filaggrin and caspase-14, were also downregulated in differentiated NLRP10 KO HSEs compared to WT HSEs (Fig. 2G). Barrier genes *FLG* and *LOR*, tight junction protein *CLDN4* and differentiation marker *KRT10* within HSEs were downregulated (Supplementary Fig. 3C). These data support a defect in keratinocyte differentiation in the absence of NLRP10.

**Fig. 3:**
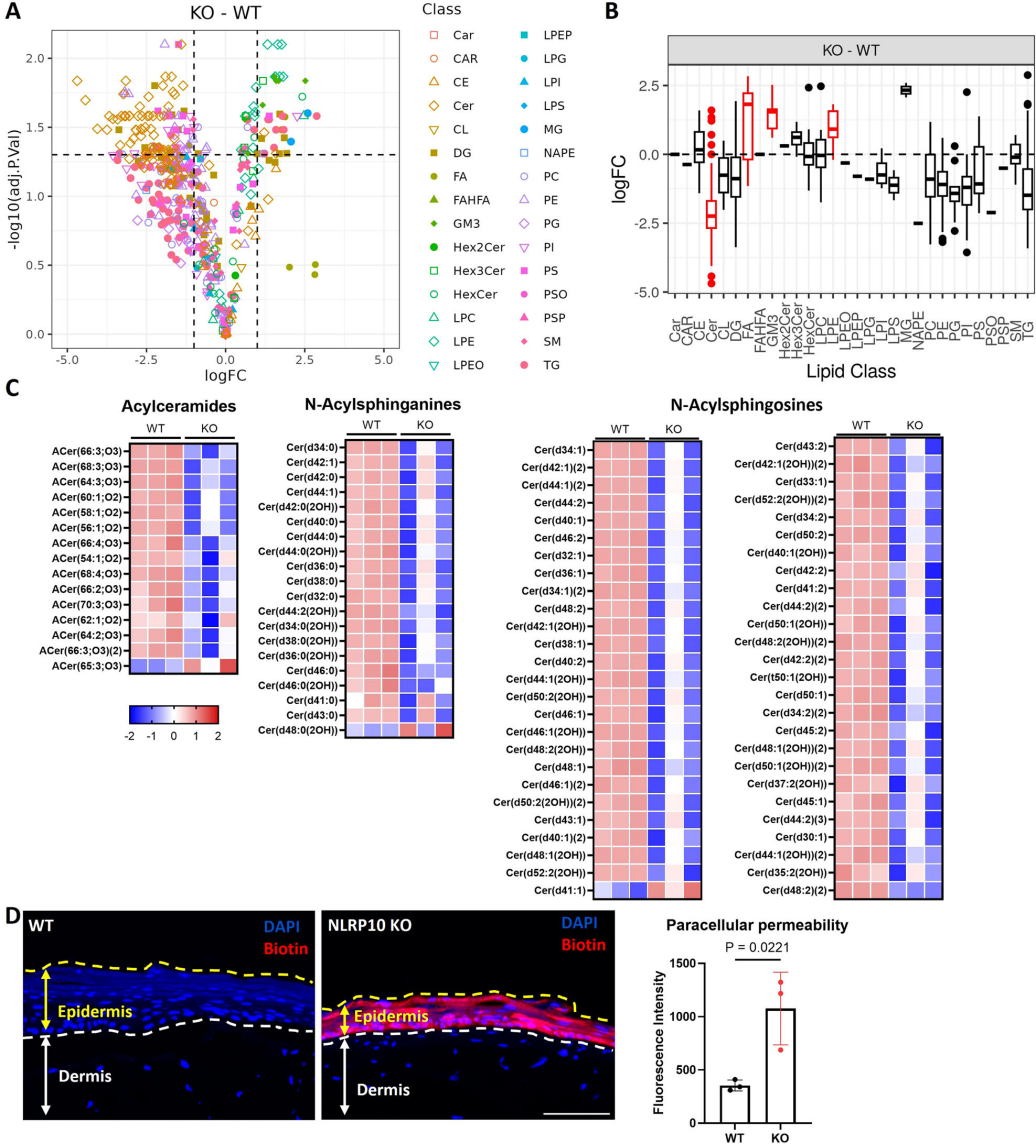
NLRP10 augments epidermal barrier function. **A** Volcano plot of lipid species differentially detected in NLRP10 KO compared to WT HSEs. **B** Boxplot of log2 fold change (logFC) of different lipid classes in NLRP10 KO compared to WT HSEs. Red color highlights statistically significantly changed lipid classes (*P* < 0.005). **C** Heatmaps from WT and NLRP10 KO HSE (*n* = 3) lipidomics analysis of the ceramide subclasses (acylceramides, N-acylsphinganines, N-acylsphingosines) included in the Cer category in the lipid set enrichment analysis in (**B**). Heatmap intensity is colored by *z*-Score for each lipid species. **D** Representative images and quantification of biotin signal intensity in HSEs with topical biotin treatment (*n* = 3). Biotin was added on top of the HSEs on the 9th day of air-lift for 45 min and washed with PBS. Frozen sections of HSE were stained with streptavidin conjugated with Alexa Fluor 594. Dashed lines highlight the epidermis region within the HSEs. Scale bar: 20 µm. Data are representative of two independent experiments and data represent mean $\pm$ S.D. and statistical significance was determined using two tailed student *t*-test.

Given that keratinocyte differentiation into stratum corneum is critical for the establishment of epidermal barrier, we next evaluated if NLRP10 deficiency affected epidermal barrier function. Lipid matrix plays a crucial role in the formation of skin barrier by preventing excessive water loss and creating a permeability barrier to avoid environmental stimulants [34]. The lipid matrix is an intercellular mix of lipids, composed primarily of ceramides, such as the acylceramide and CerEOS (a combination of esterified ω-hydroxy fatty acids and sphingosines), serving to maintain epidermal barrier integrity [35, 36]. To determine whether NLRP10 was required to maintain a normal lipid profile in the epidermis, we performed untargeted lipidomics analysis of the HSEs generated from both WT and NLRP10 KO NHEKs. Strikingly, NLRP10 KO HSEs displayed profound reduction in ceramide species from multiple sub-classes, including acylceramides, N-acylsphinganines and N-acylsphingosines. (Fig. 3A–C and Supplementary Fig. 3D). We also observed upregulation of lysophosphatidylethanolamines (LPE), gangliosides, and certain free fatty acids in NLRP10 KO HSEs (Fig. 3B and Supplementary Fig. 3D–G). The depletion of a wide range of ceramides in NLRP10 KO HSEs suggests that the barrier function may be impaired by NLRP10 deficiency. To further assess the skin barrier function directly, we topically applied biotin onto the surface of the HSEs and examined the paracellular permeability as an indicator of impaired barrier function. WT HSEs did not retain biotin expression, indicating an intact epidermal barrier (Fig. 3D). In contrast, a substantial amount of biotin was detectable in the epidermal layer of NLRP10 KO HSEs (Fig. 3D). These data were consistent with the epidermal differentiation defects in NLRP10 KO HSEs, demonstrating a critical role of NLRP10 in promoting epidermal differentiation and maintaining the epidermal barrier function.

### NLRP10 limits keratinocyte cell death

**Fig. 4:**
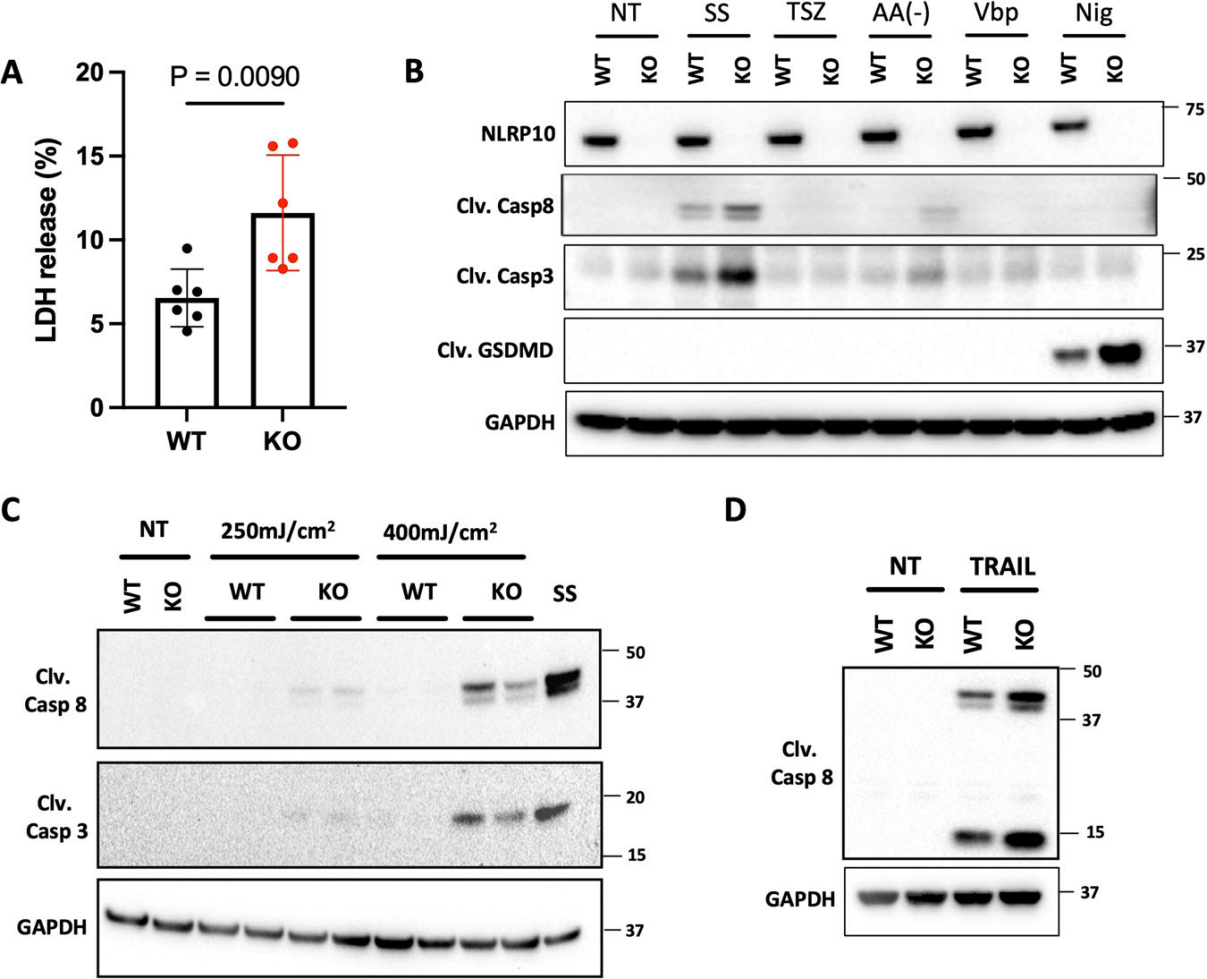
NLRP10 prevents keratinocyte cell death. Cell death monitored by LDH release assay (**A**) and immunoblotting (**B**–**D**). **A** LDH release from WT or NLRP10 KO NHEKs was measured (*n* = 6). Data represent mean $\pm$ S.D. and statistical significance was determined using two tailed student *t*-test. **B** WT and NLRP10 KO NHEKs treated with staurosporine (SS, 1 µM, 4 h), TNFα (20 ng/ml, 7 h)/SM-164 (100 µM, 7 h)/zVAD (20 µM, 7.5 h) (TSZ), amino acids deprivation (AA-, 1 h), VbP (2 µM, 4 h) or nigericin (Nig, 6.7 µM, 4 h) were analyzed by western blot with indicated antibodies. Clv. Casp8, cleaved caspase-8; Clv. Casp3, cleaved caspase-3; Clv. GSDMD, cleaved Gasdermin D. **C** WT and NLRP10 KO NHEKs incubated for 6 h in NHEK complete medium after UVB exposure (250 mJ/cm^2^ or 400 mJ/cm^2^) were analyzed by western blot with indicated antibodies. WT NHEKs were stimulated with staurosporine (SS, 1 µM, 4 h) as a positive control. **D** WT and NLRP10 KO NHEKs treated with TRAIL (300 ng/ml, 2.5 h) were analyzed by western blot with indicated antibodies. GAPDH was used as a loading control and all data are representative of more than three independent experiments.

To understand how NLRP10 regulates keratinocyte differentiation and function, we first carefully examined undifferentiated WT and NLRP10 KO NHEKs. A noticeable increase in cell death measured by lactate dehydrogenase (LDH) release was observed in NLRP10 KO NHEKs compared to WT NHEKs in the absence of any stimulation (Fig. 4A). Cell death can be triggered through distinct processes, which may have different consequences in tissue homeostasis and organismal fitness [37]. To determine which cell death pathway was involved, we treated both wild type (WT) and NLRP10 KO NHEKs with stimuli upstream of various cell death pathways. Upon staurosporine treatment or amino acid starvation, NLRP10 KO NHEKs showed increased expression of cleaved caspase-3 and cleaved caspase-8 compared to WT NHEKs, indicating a higher sensitivity to the induction of apoptosis in the absence of NLRP10 (Fig. 4B). We did not detect upregulation of phosphorylated MLKL (p-MLKL) upon treatment with TNF + SM-164+zVAD (TSZ) (Supplementary Fig. 4A), suggesting that these cells were resistant to TSZ-induced necroptosis. The inflammasome activator nigericin induced a stronger pyroptosis in NLRP10 KO NHEKs compared to WT NHEKs, as measured by enhanced cleavage of gasdermin D (GSDMD) (Fig. 4B), indicating a suppressive role of NLRP10 in nigericin-induced inflammasome-mediated pyroptosis. We were also able to detect caspase-1 cleavage in nigericin-treated WT and NLRP10 KO NHEKs (Supplementary Fig. 4A). However, the amount of cleaved caspase-1 was comparable in both genotypes (Supplementary Fig. 4A), suggesting that an additional inflammasome-activated protease was responsible for the increased GSDMD cleavage in NLRP10 KO NHEKs. Consistent with reports showing that Val-boroPro (VbP) induces pyroptosis with slow kinetics [38, 39], we were unable to detect VbP-induced GSDMD cleavage at 4 h post treatment but observed more cell death in NLRP10 KO compared to WT NHEKs at 24 h timepoint (Supplementary Fig. 4B), suggesting NLRP10 also inhibited VbP-induced pyroptosis. To further confirm the role of NLRP10 in NHEK apoptosis, we exploited additional stimulations including UV radiation and TNF-related apoptosis-inducing ligand (TRAIL). Consistently, NLRP10 KO NHEKs were more susceptible to UV-induced apoptosis (Fig. 4C). Importantly, treatment with TRAIL, a TNF superfamily protein that triggers apoptosis through caspase-8-mediated extrinsic pathway, caused higher caspase-8 activation in NLRP10 KO compared to WT NHEKs (Fig. 4D).

**Fig. 5:**
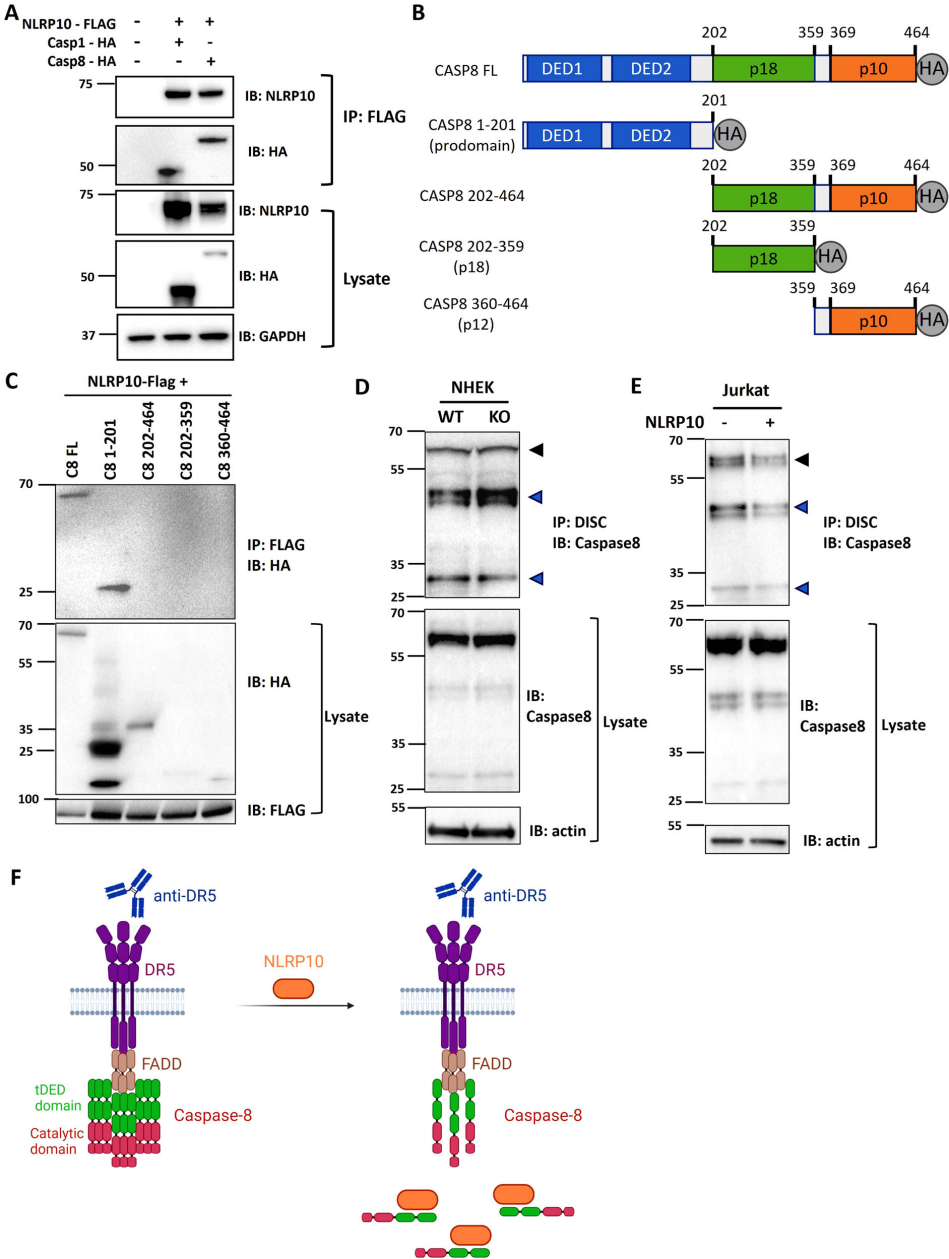
NLRP10 inhibits caspase-8 activation. **A** Co-immunoprecipitation (co-IP) of NLRP10-Flag with caspase-1-HA and caspase-8-HA in 293 T cells. Co-IP elution and cell lysate was analyzed by Western blotting using anti-HA and anti-NLRP10 antibodies. GAPDH was used as a loading control. **B** Construct design of different caspase-8 plasmids. **C** Caspase-8 constructs shown in (**B**) were co-transfected with NLRP10-FLAG in 293 T cells, and protein complex was pulled down using anti-FLAG resin and analyzed by Western blotting. **D** Western blot analysis of DR5 DISC IP carried out in WT and NLRP10 KO NHEK cells showing increased cleaved caspase-8 p43/p41 form in the DISC of NLRP10 KO NHEKs. **E** Western blot analysis of DR5 DISC IP carried out in Jurkat cells without or with NLRP10 overexpression showing decreased full length and cleaved caspase-8 in the DISC of NLRP10-overexpressing cells. The DR5 DISC IP was performed 1 h after addition of beads coupled with an agonist anti-DR5 antibody. β-actin was used as a loading control. Black arrowheads indicate full length caspase-8, and blue arrowheads represent cleaved caspase-8 in the DISC. DISC: death inducing signaling complex. **F** Illustration of DR5 DISC in the absence or presence of NLRP10. Agonistic anti-DR5 antibody induces the assembly of DISC and recruitment of caspase-8 to the complex. NLRP10 interacts with the DED domain of caspase-8, reduces caspase-8 density recruited to the DISC and thereby suppresses caspase-8 activation. Caspase-8 is color-coded to highlight the tandem DED (tDED) domain in green and catalytic domain in red. DR5, death receptor 5; FADD, Fas-Associated Death Domain Protein; DED, death effector domain.

To investigate the mechanism by which NLRP10 inhibits apoptosis, we performed co-immunoprecipitation (co-IP) experiments of NLRP10 and the effector protein caspase-8. As caspase-1 was reported to interact with NLRP10 [16], it was used as a positive control. NLRP10 was able to efficiently pull down both caspase-1 and caspase-8 (Fig. 5A). We further mapped the domains of each caspase that interacted with NLRP10. Consistent with a previous report [16], NLRP10 interacted with the catalytic domain of caspase-1 (Supplementary Fig. 4C, D). In contrast, NLRP10 bound to the pro-domain of caspase-8 consisting of two tandem death-effector domains (tDEDs) (Fig. 5B, C). DED domains are involved in caspase-8 recruitment to the death-inducing signaling complex (DISC) and its subsequent oligomerization and activation by autocleavage [40]. We then assessed whether NLRP10 affects caspase-8 activity by impeding its recruitment to the DISC. To this end, we utilized Dynabeads coupled with an agonistic anti-DR5 antibody to induce the formation of the DR5 DISC and affinity purified the complex from WT and NLRP10 KO NHEKs [41]. DR5 DISC from NLRP10 KO NHEKs contained more cleaved p43/p41 caspase-8 compared to WT NHEK, with similar levels of the full-length caspase-8 (Fig. 5D). To further confirm the effect of NLRP10 on caspase-8 activity, we performed DR5 DISC IP experiment in Jurkat cells with or without NLRP10 overexpression. Overexpression of NLRP10 drastically reduced the amount of caspase-8 in both its full length and cleaved forms in the DISC (Fig. 5E), confirming that NLRP10 decreases the caspase-8 density recruited to the DISC and thereby suppresses DISC-mediated caspase-8 activation (Fig. 5F).

### NLRP10 enhances p63 signaling in keratinocytes

**Fig. 6:**
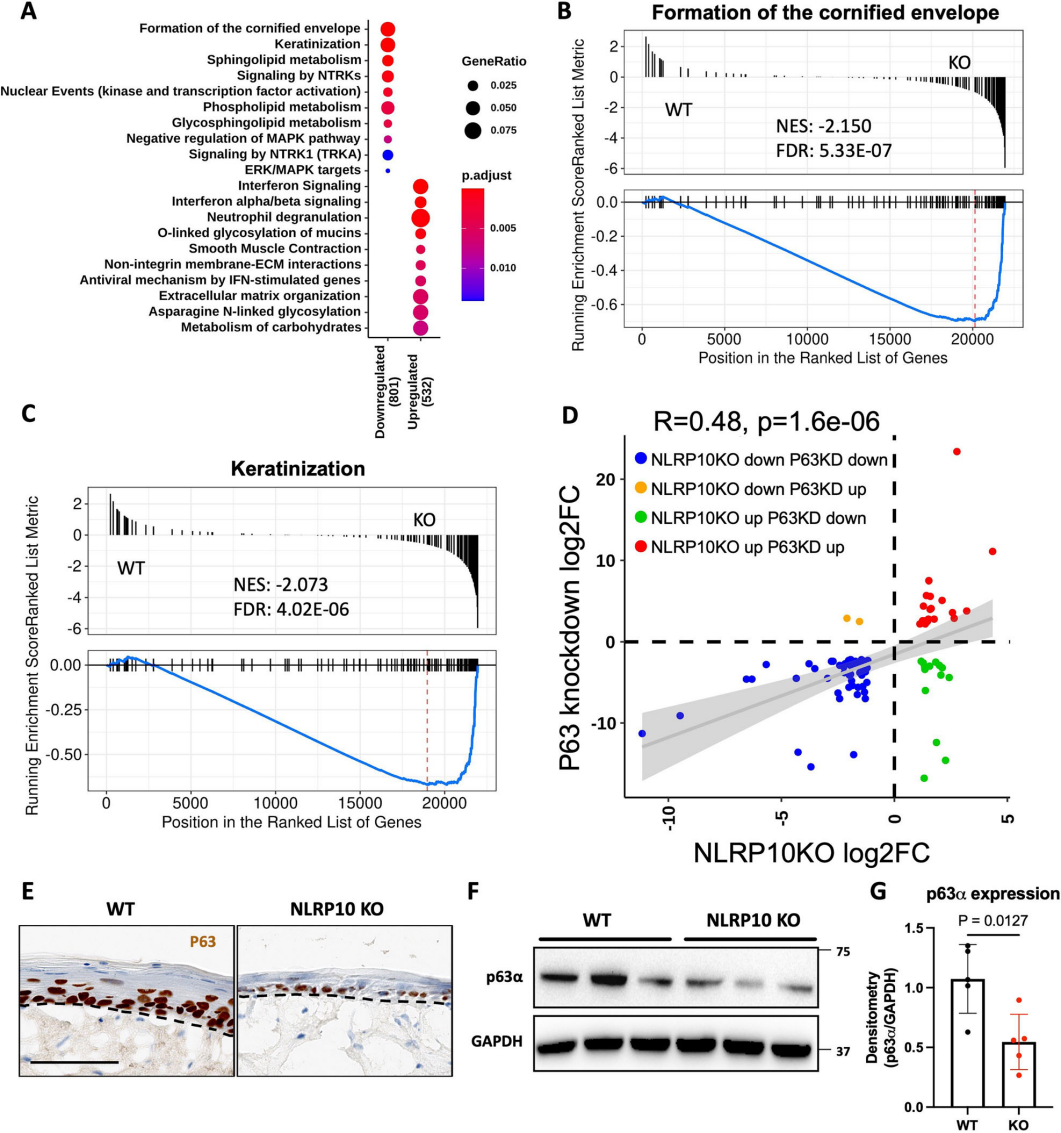
NLRP10 enhances p63 signaling in keratinocytes. **A** Dot plot of enriched reactome pathways downregulated and upregulated in NLRP10 KO HSEs. **B**, **C** Gene set enrichment plots for top reactome pathways are shown: Formation of the cornified envelope (**B**) and keratinization (**C**). **D** scatter plot showing common genes significantly changed (adjusted *P* < 0.05) between NLRP10 KO (this study) and p63 knockdown (Truong et al.) [42]. Correlation coefficient (R) and *p*-value are shown. **E** Immunohistochemistry staining of p63 in WT and NLRP10 KO HSEs. Dashed lines highlight the boundary between the epidermis and the dermis within the HSEs. **F**, **G** Western blot of p63α in WT and NLRP10 KO NHEK (*n* = 5). Quantification was performed with densitometry normalized to housekeeping gene GAPDH.

To elucidate the function of NLRP10 during keratinocyte differentiation, we performed transcriptome analysis on WT and NLRP10 KO HSEs. We observed significant gene expression changes between NLRP10 KO and WT HSEs (Supplementary Fig. 5A). Gene set enrichment analysis revealed formation of the cornified envelope and keratinization as the most downregulated pathways (Fig. 6A–C and Supplementary Fig. 5B, C). Sphingolipid metabolism, phospholipid metabolism and glycosphingolipid metabolism pathways were also significantly reduced in NLRP10 KO HSEs (Fig. 6A and Supplementary Fig. 5D–F), in agreement with reduced ceramides in NLRP10 KO HSEs (Fig. 3C).

The defects of epidermal differentiation and barrier function in NLRP10 KO HSEs highly resembled the phenotype of p63-deficient human epidermis [42]. Given that p63 is considered a master regulator of epidermal differentiation [43–45], we investigated whether p63 signaling was affected by the loss of NLRP10. To this end, we interrogated our RNAseq dataset to identify genes regulated by p63 during keratinocyte differentiation [42]. Gene expression pattern of p63 regulated genes highly coincided with the changes exhibited in NLRP10 KO cells, *i.e*. genes downregulated in p63 knockdown cells were also largely downregulated in NLRP10 KO cells and vice versa (Fig. 6D and Supplementary Fig. 6A, B). This highlights a potential link between NLRP10 and p63 signaling in keratinocytes. We then attempted to measure the mRNA and protein levels of p63 in NLRP10 KO cells. Transcript level of *TP63*, the gene that encodes p63 protein, was not affected in NLRP10 KO HSEs (Supplementary Fig. 6C). To evaluate p63 protein levels, we performed p63 IHC in WT and NLRP10 KO HSEs. There was a reduction in p63 IHC staining and western blot analysis revealed significant downregulation of p63 protein (Fig. 6E–G). We also confirmed the downregulation of p63 direct target genes *CHAC1* and *SCT2* by qPCR (Supplementary Fig. 6D). p63 mediates terminal epidermal differentiation by regulating key downstream target genes expression including IKKα [46, 47] and the ZNF750-KLF4 axis [48]. IKKα transcript and protein levels were similar in WT and NLRP10 KO HSEs (Supplementary Fig. 6E, F), suggesting that NLRP10 regulates epidermal differentiation independent of IKKα. Interestingly, we observed a significant reduction of *ZNF750* and *KLF4* expression in NLRP10 KO HSEs (Supplementary Fig. 6G), indicating that the NLRP10-p63-ZNF750-KLF4 axis might be crucial in directing epidermal differentiation. Furthermore, we assessed p63 expression in the skin of healthy control and AD patients. Despite the presence of epidermal hyperplasia and intercellular edema, the p63 staining pattern in AD patient samples resembled that of healthy control samples (Supplementary Fig. 6H). Given that AD is frequently accompanied by an increase in type 2 inflammation, which affects p63 levels [49, 50], the clinical phenotype observed in AD reflects the combined effects of keratinocyte-intrinsic changes and immune-related influences and may therefore differ from our observations in HSEs that only reflect keratinocyte-intrinsic changes.

### NLRP10 stabilizes p63

**Fig. 7:**
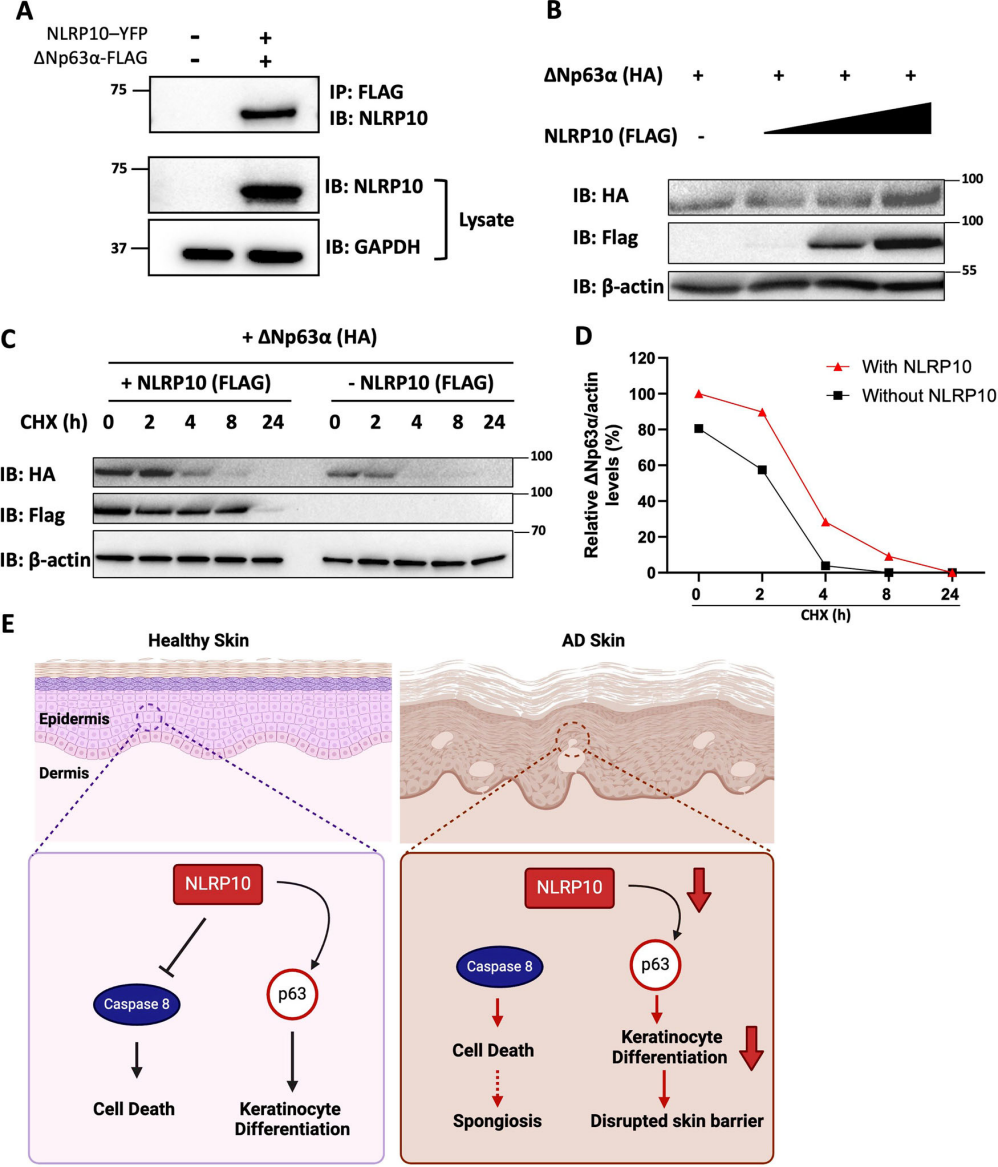
NLRP10 stabilizes p63 in keratinocytes. **A** Co-immunoprecipitation of NLRP10-YFP and $\triangle$Np63α-FLAG in 293 T cells. GAPDH was used as a loading control. **B** COS-7 cells transfected with $\triangle$Np63α-HA and increasing doses of NLRP10-FLAG were subjected to cell fractionation and immunoblotted with antibodies to the indicated proteins. β-actin was used as a loading control. Samples were prepared 48 h after transfection. **C** COS-7 cells transfected with $\triangle$Np63α-HA with or without NLRP10-FLAG for 48 h were incubated with cycloheximide (CHX) for up to 24 h, and analyzed by immunoblotting for $\triangle$Np63α-HA, NLRP10-FLAG and β-actin (loading control) levels. **D** Relative level of the ratio of $\triangle$Np63α-HA to β-actin in (**C**). The immunoblot band was analyzed by ImageJ software. **E** Proposed working model of NLRP10. NLRP10 inhibits caspase-8-mediated cell death and stabilizes p63 to promote keratinocyte differentiation and formation of skin barrier in healthy skin. In AD skin, NLRP10 expression is reduced. With decreased NLRP10 level, keratinocytes are more susceptible to cell death and have reduced differentiation, which altogether lead to spongiosis and disrupted skin barrier.

Next, we investigated the mechanism of how NLRP10 regulates p63. Transcript level of p63 was not affected (Supplementary Fig. 6C), indicating that NLRP10 regulates p63 at the post-transcriptional level. We then studied whether NLRP10 interacts with p63 and affects its protein stability. We co-expressed NLRP10 and ΔNp63α, the predominant p63 isoform expressed in the skin [42, 51], in 293 T cells and performed co-immunoprecipitation. The results showed that NLRP10 could indeed form a complex with ΔNp63α (Fig. 7A). To evaluate whether NLRP10 increases the stability of p63, we co-transfected cells with ΔNp63α-expressing plasmid together with increasing concentrations of NLRP10-encoding plasmid. Increased expression of NLRP10 led to higher expression of ΔNp63α (Fig. 7B). Furthermore, we chased cells with cycloheximide (CHX) to inhibit protein synthesis and measured ΔNp63α degradation over time in the presence or absence of NLRP10. Addition of NLRP10 greatly enhanced ΔNp63α expression and delayed its degradation (Fig. 7C, D). Thus, NLRP10 likely regulates the protein stability of p63 through protein-protein interaction. These results together support important roles of NLRP10 in preventing stress-induced keratinocyte cell death via inhibiting caspase-8 and enhancing keratinocyte differentiation and barrier function through stabilization of the epidermal master regulator p63 (Fig. 7E). Loss of NLRP10 in AD might contribute to enhanced susceptibility to cell death, decreased keratinocyte differentiation and ultimately increased spongiosis and disruption of the skin barrier.

## Discussion

Currently most AD therapeutic strategies focus on dampening immune response [2]. However, given that epidermal barrier disruption also contributes to the pathogenesis of AD [1, 3], it might be more effective to manage the disease by incorporating factors that promote epidermal barrier function into current therapeutic strategy. In this study, we showed NLRP10 is downregulated in AD, suggesting a key role of NLRP10 in AD pathogenesis. We further characterized the critical role of NLRP10 in maintaining epidermal homeostasis through two mechanisms. First, NLRP10 protects keratinocytes from cell death by inhibiting caspase-8 activity. Second, NLRP10 promotes keratinocyte differentiation and reinforces barrier function by stabilizing p63 expression. This dual functionality of NLRP10 emphasizes its significance in maintaining normal skin homeostasis, providing insights into potential therapeutic interventions to restore skin barrier function and homeostasis in AD patients.

The outermost stratum of the epidermis, termed the stratum corneum (SC), is formed by terminally differentiated keratinocytes (also known as corneocytes). The SC is composed of a protein and lipid enriched cornified envelope and is responsible for the establishment of epidermal barrier function [52]. Filaggrin is a major structural protein of the cornified envelope and plays important roles in the formation of the cornified envelope by aggregating keratin fibers into bundles and thereby collapsing the corneocytes into flattened discs [3]. Filaggrin also contributes to skin moisturization through break-down into natural moisturizing factors [3]. Lipids released into the intercellular space during keratinocyte terminal differentiation spontaneously organize into multiple layers between the SC cells and form the permeability barrier [53]. The epidermal barrier in the SC limits passive water loss, reduces the penetrance of allergens and irritants and prevents microbial infection [53]. Utilizing an in vitro human skin equivalent model, we found that NLRP10 is critical for proper keratinocyte differentiation and expression of filaggrin and other important molecules essential to the formation of cornified envelope. Lipidomics and transcriptomics analysis revealed that NLRP10 is essential for the production of ceramides. Ceramides are the major lipid constituent of lamellar sheets present in the intercellular spaces of the stratum corneum and play an essential role in structuring and maintaining the water permeability barrier function of the skin [54]. These findings support a key role of NLRP10 in controlling keratinocyte differentiation and facilitating the formation of epidermal barrier function.

Keratinocyte apoptosis has been suggested to contribute to the formation of eczema and spongiosis, and is mostly seen in acute and subacute lesions [55]. Skin-infiltrating T cells in AD induce keratinocyte apoptosis, leading to subsequent cleavage of E-cadherin and resisting desmosomal cadherins, which promotes spongiosis formation [56, 57]. Activated skin infiltrating T cells can trigger keratinocyte apoptosis through FasL-Fas pathway by cell-cell contact [58] or the release of proapoptotic cytokines including IFNγ, TNFα and TNF-like weak inducer of apoptosis (TWEAK) [59, 60]. It has been reported that TRAIL is also upregulated in AD samples [61], suggesting that multiple signaling pathways can lead to keratinocyte apoptosis in AD. We found that NLRP10 prevents keratinocyte apoptosis induced by various apoptotic stimuli by inhibiting the activation of caspase-8 (Fig. 2), highlighting a protective role of NLRP10 in promoting keratinocyte survival under stress conditions. Upon the ligation of death receptors, FAS-associated death domain protein (FADD) binds to the intracellular death domains of trimerized death receptors (DRs) and exposes the DED domain to recruit procaspase-8 to form the death-inducing signaling complex (DISC) [40]. The caspase-8 tandem DED then assembles into filaments through quasi-equivalent contacts, leading to caspase-8 activation [62]. Caspaspe-8 activation can be regulated at the level of DISC assembly. Recent cryo-EM structural analysis suggests that cellular FLICE (FADD-like IL-1β-converting enzyme)-inhibitory protein short form (c-FLIPs) binds caspase-8 via DED interactions and comingles with caspase-8 filaments to reduce the local concentration of caspaspe-8 [62]. It has also been suggested that the interaction between c-FLIPs and caspase-8 blocks caspase-8 filament elongation by steric hinderance of the canonical tandem DED binding site [63]. NLRP10 forms a complex with the tandem DED domain of caspase-8 and interferes with DED-mediated recruitment of caspase-8 to the DISC (Fig. 3F). It remains interesting to determine if NLRP10 inhibits caspase-8 activation through a mechanism similar to c-FLIPs and whether other proteins are involved in the regulation process.

p63 is a master regulator of epidermal proliferation and differentiation [43–45]. Loss of the epidermal isoform ΔNp63α post lineage commitment results in tissue hypoplasia and differentiation defects [42, 47]. NLRP10 KO keratinocytes exhibit similar differentiation defects and gene signatures compared to p63 knockdown, suggesting that regulation of p63 might be the key link to the phenotype observed in NLRP10 KO HSEs. Indeed, we detected downregulation of p63 at the protein level but not at the transcriptional level, pointing to a post-transcriptional regulation of p63 by NLRP10. p63 regulates dynamic gene expression during epidermal differentiation [64, 65]. Several important regulators of terminal epidermal differentiation have been identified as direct targets of p63, including IKKα [46, 47] and ZNF750 [48]. We observed downregulation of p63 target gene *ZNF750*, but not *IKKα*, suggesting that NLRP10 modulates terminal epidermal differentiation through an IKKα-independent mechanism. ZNF750 is one of the earliest transcription factors activated by p63 during epidermal differentiation [48]. Overexpression of ZNF750 is sufficient to rescue terminal differentiation defects caused by p63 loss [48]. ZNF750 also controls KLF4 expression, an effector of terminal differentiation, to drive the differentiation gene expression [48, 66]. In accordance with a decrease in *ZNF750* expression, *KLF4* expression was also significantly reduced in the absence of NLRP10, indicating that the NLRP10-p63-ZNF750-KLF4 axis plays a crucial role in directing terminal epidermal differentiation. Regulation of p63 may occur by proteasome-mediated protein degradation driven by the interaction with multiple E3 ubiquitin ligases [67]. Of note, p63 protein level can be balanced by stabilization through interaction with a cytosolic scaffold protein STXBP4 and destabilization via RACK1 [68]. Interestingly, p63 may also be regulated at the level of cytoplasm-to-nucleus transport [69] and cytoplasmic localization of p63 has also been reported [70, 71]. We identified NLRP10 as another important regulator of p63 in keratinocytes. NLRP10 interacts with p63 and extends its protein stability and half-life. It is possible that interaction with NLRP10 blocks the accessibility of E3 ligases to p63 for targeted degradation. Given that p63 can be targeted by multiple E3 ligases, the exact mechanism by which NLRP10 protects p63 from degradation warrants further investigation.

One limitation of our study is that the immune system-mediated inflammatory responses were not modeled in our system. Considering that AD is a complex disease with a strong component of type 2 immune response and severe itch-related neuroinflammation [1, 5], which contribute to the clinical presentation of epidermal hyperplasia and the molecular regulation of p63 [49, 50, 72, 73], it would be of great interest to further investigate the impact of modulating NLRP10 in a disease-relevant context.

In summary, our data characterize an important role of NLRP10 in promoting keratinocyte survival and epidermal barrier function, which are frequently altered in inflammatory skin diseases such as AD. Therapeutic targeting NLRP10 might represent an attractive approach to restore epidermal functions in diseased conditions, which would complement current immunomodulatory therapies to achieve clinical benefits.

## Methods

### Ethics approval and consent to participate

All methods included in this study were performed in accordance with the relevant guidelines and regulations. Studies involving clinical samples have been approved by Sterling IRB. No studies with live vertebrates were performed.

All human specimens were collected via Amgen Human Tissue Science Center study specifications under site-specific Institutional Review Board or Ethics Board approval with appropriate informed consent in compliance with all applicable laws and regulations. In all cases, materials obtained were surplus to standard clinical practice and standards of care. Patient identity, Protected Health Information (PHI) and other identifying information were redacted from tissues and clinical data prior to submission to Amgen.

### Immunohistochemistry

Several positive control cell lines and negative control cell lines were used to develop an immunohistochemistry (IHC) assay for NLRP10. Positive control cell lines included Chinese hamster ovary (CHO-K1) cells stably expressing human NLRP10, Normal Human Epidermal Keratinocyte (NHEK), a cell line that is known to express low to intermediate levels of human NLRP10 protein, and Normal Human Dermal Fibroblast (NHDF), a cell line that is known to express intermediate to high levels of human NLRP10 protein. Negative control cell lines included parental CHO-K1 cells and human NLRP3-transfected CHO-K1 cell lines. Cell pellets containing ~5 × 10^7^ cells were fixed in 10% neutral buffered formalin (NBF), suspended in Histogel containing agarose/glycerin (Thermo Fisher Scientific, HG-4000-12), and processed to paraffin blocks. In addition, normal human formalin-fixed paraffin embedded (FFPE) skin was used as a positive control tissue in assay development.

Normal and AD FFPE samples were sectioned at 4 μm and mounted on positive charged glass slides. Fully automated IHC assay was performed on a Discovery Ultra stainer (Ventana Medical Systems, Tucson, AZ). The slides were baked on the Discovery Ultra stainer at 60 °C for 8 min, de-paraffinized with Ventana Discovery wash (Ventana, 950–510) for 3 cycles (8 min each) at 69 °C and underwent cell conditioning (target retrieval) at 95 °C for 32 min in CC1 buffer (Roche/Ventana, 950-500). Slides were first incubated with Background Sniper (Biocare Medical, BS966M) in a Ventana Option Prep kit (Ventana, 771-751) and Discovery Inhibitor (Roche, 760-4840). Multiplex panels were developed with sequential antibody incubation followed by denaturation steps at 95 °C with CC2 (Roche, 950-223) for 8 min. The first panel has NLRP10 (Sigma-Aldrich, MABC293, 10 µg/ml on slide) stained with purple chromogen (Roche, 760-229) followed by Cytokeratin14 (Thermofisher, MA5-11599, 0.2 µg/ml on slide) stained with teal chromogen (Roche, 760-247). The second panel has ZO-1 (Invitrogen, 33-9100, 1 µg/ml on slide) stained with purple chromogen followed by Filaggrin (Abcam, ab221155, 0.543 µg/ml on slide) stained with teal chromogen. All antibodies were diluted with Ventana diluent with Casein (Ventana, 760-219) in a Ventana Prep kit (Ventana, 770-001) or with a matched concentration of isotype control Rat IgG2a,k antibody (Invitrogen, 14-4321-82, 10 µg/ml on slide). For the single P63 IHC DAB staining, p63 antibody (Roche, 790-4509, 0.035 µg/ml on slide, ready to use) was used with the Roche ready-to-use protocol. Following antibody addition, slides were incubated with either Ventana anti-Rabbit HQ (Ventana, 760-4814) or Ventana anti-Mouse HQ for 24 min at RT, then Ventana anti-HQ HRP (Ventana, 760-4820) for 24 min at RT, and signals detected with Ventana chromogen. Slides were counterstained with Ventana Hematoxylin ll (Ventana, 5266726001) for 4 min and Ventana bluing reagent (Ventana, 526676900) for 4 min. Slides were removed from the Discovery Ultra stainer and washed in Dawn soap water after staining was completed. Slides were dehydrated and cover slipped on Leica Spectra coverslipper (Leica Biosystems Inc, SN 02000926). All slides were scanned into Aperio Leica GT450 (Leica Biosystems Inc.) and images were uploaded to Concentriq (Proscia Inc.). Digital images were reviewed by a board-certified veterinary pathologist. The degree of acanthosis, presence of spongiosis, and severity of inflammation was evaluated on H&E-stained sections, obtained from Cureline BioPathology LLC (Brisbane, CA). The staining distribution, location, and intensity of each IHC chromogen was evaluated in comparison with normal tissue.

### Plasmids, cell lines and cell culture

Normal human epidermal keratinocytes (pooled donors, C-12005) and normal human dermal fibroblasts (C-12300) were purchased from PromoCell and cultured under standard conditions (37 °C and 5% CO2) in Keratinocyte Growth Medium 2 (PromoCell, C-20011) and fibroblast growth medium (PromoCell, C-23110), respectively. NHEKs were maintained in medium with 10 μM Y-27632 (Sellek Chem, S1049).

HEK293T cells (Takara, 632180) and COS-7 (ATCC, CRL-1651) cells were cultured in Dulbecco’s modified Eagle’s medium (DMEM) (Gibco, 10569-010) supplemented with GlutaMAX (Gibco, 35050-061), penicillin/streptomycin (Gibco, 15140-122) and 10% fetal bovine serum (FBS) under standard conditions (37 °C and 5% CO2).

NLRP10-FLAG, Caspase-HA and p63-HA constructs were synthesized by Genscript. All constructs were in pcDNA3.1 backbone vector.

### Generation of NLRP10 KO cell line

Two guide RNAs targeting the coding regions right after the ATG start codon of the *NLRP10* gene were designed with Benchling. The sequences of the gRNAs are shown in the Table S1. Transfection was performed using Amaxa Human Keratinocytes Nucleofactor kit (LPD-1002) according to the manufacturer’s recommendation. Briefly, 1 × 10^6^ NHEKs were used in each reaction mixed with Cas9-gRNA ribonucleoprotein complexes. Knockout was confirmed by Western blotting. KO pool was used throughout the study to minimize clonal bias.

### LDH cytotoxicity assay

2 × 10^5^ NHEKs were seeded in 100 μl of NHEK complete medium per well in a 96-well plate and incubated for overnight. 50 μl of the harvested supernatant was used to measure LDH release based on the manufacturer’s protocol (CyQuant LDH Cytotoxicity assay, ThermoFisher, C20300). THP-1 cells treated with 1 μg/ml LPS 1 h prior to 5 μM nigericin treatment for 4 h were used as a positive control.

### Cell death assay

1.5 × 10^6^ NHEKs were seeded in each well in a 6-well plate. 1 μM staurosporine (Sigma, 569396) treatment for 4 h was used to induce apoptosis. To induce necroptosis, cells were pretreated with 20uM Z-VAD (MCE, HY-16658B) for 30 min prior to adding 20 ng/ml TNF-α (R&D, 210-TA) and 100 nM SM-164 (MCE, HY-15989) for 7 h. To induce autophagic cell death, NHEKs were animo acid starved for 1 h in HBSS. NHEKs were also treated with 2 μM VbP (Talabostat mesylate, Apexbio Technology, Catalog No. B3941) for 4 h or 6.7 μM Nigericin (Sigma, SML1779) for 4 h to induce pyroptosis. For additional apoptosis assay, NHEKs were treated with 300 ng/ml human recombinant TRAIL (R&D, 375-TL-010) for 2.5 h. NHEK complete medium was replaced with 500 μl of PBS per well in a 6-well plate before UVB treatment (250 mJ/cm^2^ or 400 mJ/cm^2^, Thomas Scientific, CL-3000). After UVB treatment, NHEK complete medium was added for another 6 h of incubation. The NHEK cell lysates from each condition were harvested and used for Western blotting. The primary antibodies used in the assay are listed in the Table S2.

### Western blotting and immunoprecipitation (IP)

Cells were lysed in IP lysis/wash buffer (ThermoFisher, 26146) for IP or RIPA buffer (ThermoFisher, 89900) for Western blotting in the presence of a protease/phosphatase inhibitor cocktail (ThermoFisher, 78442). Protein concentrations in cell lysates were quantified using the BCA assay (ThermoFisher, 23227). 30 μg and 1 mg of total protein lysate were used for Western blotting and IP respectively. Western blotting and IP were performed by following standard manufacturers’ protocols. The immune complex was eluted with 1X FLAG peptide (Sigma, F3290). Primary antibodies were used at 1:1000 dilution at 4 °C for overnight and secondary antibodies were used at 1:10,000 dilution at room temperature for 2 h. All primary and secondary antibodies are listed in Table S2. Protein samples were visualized using a ChemiDoc XRS+ imaging system (BioRad).

### DR5 DISC assay

Conatumumab (ThermoFisher, MA5-41826), which recognizes the extracellular region of DR5, was conjugated to Dynabeads using the antibody coupling kit (Invitrogen, 14311D) as per the manufacturer’s instructions. In detail, 100 μg of Conatumumab was conjugated to 6 mg Dynabeads and resuspended with 600 μl buffer and stored at 4 °C. For the DR5 DISC assay, 30 μl of Dynabeads coated with 5 μg of Conatumumab was added to 2 × 10^6^ cells and incubated for 1 h at 37 °C. The cells were then lysed in DISC buffer (1% Triton X-100, 30 mM Tris-HCL (pH 7.5), 150 mM NaCl and 10% glycerol) on ice for 15 min. The Conatumumab-coated Dynabeads were collected and washed 5 times in DISC buffer prior to resuspension in SDS sample buffer and analysis by western blotting.

### Cycloheximide chase assay

1.25 × 10^5^ COS-7 Cell was seeded in each well in a 12-well plate. 24 h later, cells were transfected with ΔNp63α-HA together with NLRP10-Flag or pcDNA3.1 empty vector plasmid. 48 h after transfection, cells were treated with 20 μg/ml Cycloheximide (Sigma, C4859) for 2 h, 4 h, 8 h, and 24 h. Then cells were harvested and prepared for western blot.

### Human skin equivalent culture

In vitro 3D human skin equivalent tissue was constructed following manufacturer’s instructions (Sigma). Briefly, collagen bed was made up with type I rat tail collagen (Millipore Sigma, 06-115), Matrigel (Millipore Sigma, CLS356234) and 5X reconstitution buffer: 1.1% NaHCO_3_, 0.025 N NaOH, 100 mM HEPES, 5X DMEM/F12. NHDFs (4 × 10^4^ per well) were added into the collagen bed and 150 μl of the collagen bed-NHDF was added into each well of 24-well trans well plate (Costar, 3413). The collagen bed-NHDF was solidified by incubating at 37 °C for 1 h. 1 × 10^5^ NHEK in 150 μl was added on top of the collagen bed-NHDF in each well and 1 ml of the NHEK complete medium was added outside of the trans-well. On day 4, the trans-wells were air lifted by removing NHEK medium and 500 μl of the differentiation medium (Millipore Sigma, SCM310) was added outside of the trans well. The differential medium was replaced every 2 days and the skin culture was harvested after 9 days in air-lift culture.

### Human skin equivalent immunofluorescence (IF) and IHC staining

The human skin equivalent culture was fixed in 4% PFA for 3 h and went through cryoprotection with 15% sucrose for overnight and 30% sucrose for 2 h before being placed in the OCT compound and frozen. The OCT frozen section was used for IF staining. Each section was rehydrated with PBS for 10 min and permeabilized with 0.5% Triton X-100 (Sigma, AC215682500) in Tween 20-TBS (TBS-T) for 15 min. Then the sections were incubated in 10% normal goat serum for an hour, then in each primary antibody for 1 h and secondary antibodies for 1 h at room temperature. Primary antibodies and secondary antibodies used in this study are listed in Table S2.

For IHC staining on HSE organoid, OCT frozen section slides were baked at 60 °C for 20 min prior to incubation in 10% NBF for 1 h at room temperature. Slides were then washed for 10 min in 0.5% Triton X-100 in TBS-T before loading onto a Discovery Ultra stainer with no baking and deparaffinization steps. Cell conditioning (target retrieval) was performed at 95 °C for 16 min in CC1 buffer. Either Ki67 (Abcam, ab16667, 0.03 µg/ml on slide) or p63 antibody (Roche, 790-4509, 0.035 µg/ml on slide, ready to use) was added to the slide. The Ki67 antibody was diluted with Ventana diluent with Casein in a Ventana Prep kit and incubated for 1 h at room temperature. The p63 antibody was incubated for 16 min at 37 °C. Following antibody incubation, slides were incubated with Ventana anti-Rabbit HQ (Ventana, 760–4814) for 24 min at RT, then Ventana anti-HQ HRP (Ventana, 760–4820) for 24 min at RT. Signals were detected with Ventana chromogen, then slides were counterstained with Ventana Hematoxylin ll for 4 min and Ventana bluing reagent for 4 min.

### Human skin equivalent paracellular permeability assay

After 9 days of the air lift culture, 10 μl of 5 mg/ml EZ-link Sulfo-NHS Biotin (ThermoFisher, 21217) was added onto the top of the skin culture for 45 min and washed with PBS. Samples were then fixed and processed for cryo-sectioning. Biotin was detected with streptavidin conjugated to Alexa 594 (Invitrogen, S32356). Nuclei were stained with DAPI (ThermoFisher, 42248) and paracellular permeability was quantified with biotin fluorescence intensity using Image J.

### RNA-Seq library preparation and data analysis

Human skin equivalent cultures were snap frozen and sent to Azenta for standard RNA sequencing. Total RNA was extracted from frozen cell samples using Qiagen RNeasy Plus Universal mini kit following manufacturer’s instructions (Qiagen, Hilden, Germany). RNA samples were quantified using Qubit 2.0 Fluorometer (Life Technologies, Carlsbad, CA, USA) and RNA integrity was measured using the RNA Screen Tape on Agilent 2200 TapeStation (Agilent Technologies, Palo Alto, CA, USA). ERCC Ex-fold RNA reagent (Cat: 4456739) from ThermoFisher Scientific, was added to normalized total RNA prior to library preparation following manufacturer’s protocol. The RNA sequencing libraries were prepared using the NEBNext Ultra II RNA Library Prep Kit for Illumina using manufacturer’s instructions (New England Biolabs, Ipswich, MA, USA). Briefly, mRNAs were initially enriched with Oligo-d(T) beads. Enriched mRNAs were fragmented for 15 min at 94 °C. First strand and second strand cDNA were subsequently synthesized. cDNA fragments were end repaired and adenylated at 3’ends, and universal adapters were ligated to cDNA fragments, followed by index addition and library enrichment by PCR with limited cycles. The sequencing library was validated on the Agilent TapeStation (Agilent Technologies, Palo Alto, CA, USA), and quantified by using Qubit 2.0 Fluorometer (ThermoFisher Scientific, Waltham, MA, USA) as well as by quantitative PCR (KAPA Biosystems, Wilmington, MA, USA). The sequencing libraries were multiplexed and clustered on 2 lanes of a flowcell. After clustering, the flowcell was loaded on the Illumina HiSeq 4000 instrument according to manufacturer’s instructions. The samples were sequenced using a 2 × 150 Pair-End (PE) configuration.

RNA-Seq reads were aligned to human genome GRCh38 (GENCODE V24) using OSA aligner [74] of the OmicSoft Array Suite (v10.0, QIAGEN, USA). Gene level expression was quantified by RSEM [75] and normalized as Fragments Per Kilobase per Million mapped reads (FPKM). Downstream plotting and analyses were done using packages in R 4.0.4 (R Core Team). DESEQ2 (v1.30.1) [76] was used to identify the differential expressed genes in NLRP10 depleted versus control skin cultures. Only expressed genes (sum of read counts over 100) were considered in the differential expression analysis. Identified differentially expressed genes were visualized in a volcano plot by EnhancedVolcano (v3.18.1) and fed into pathway analysis by clusterProfiler (v3.18.1) [77] using gene sets from ReactomeRA (v1.34.0) [78] and Msigdbr (v7.5.1) [79]. The enriched gene sets were visualized using dotplot() and gseaplot() function. Heatmap representations of genes in enriched pathways were created by pheatmap (v1.0.12). Expression changes of p63 downstream targets (adjusted *P*-value < 0.05, fold change > 2) and NLRP10 targets (adjusted *P*-value < 0.05) were compared and visualized in a scatter plot by ggplot2 (v2.2.5).

### Lipidomics analysis

Human skin equivalent cultures were harvested and snap frozen. Untargeted lipidomics was performed by Cayman Chemical as described below. Briefly, 200 μl PBS solution was added to each sample, and samples were transferred to Precellys 0.5 mL lysing kit VK05 tubes, then homogenized using a Precellys Evolution Homogenizer for 3 cycles of 30 s at 7200 rpm, with 60 s pauses between cycles. The homogenate was transferred to 8 mL glass tubes for lipid extraction, where 4 mL methyl tert-butyl ether (MTBE), 1.2 mL methanol, and 10 μl of an internal standard mix were sequentially added to each sample. After vigorous vortexing, the tubes were placed on a tabletop shaker at 500 rpm at room temperature for 1 h. Phase separation was then induced by the addition of 1 mL water and then centrifuged at 2000 × g for 20 min. The upper organic phase of each sample was carefully removed using a Pasteur pipette and transferred into a clean glass tube. The remaining aqueous phase was reextracted with 1.5 mL of the upper phase of an MTBE/methanol/water 10:3:2.5 (v/v/v) mixture. After vortexing and centrifuging, the organic phase was collected and combined with the initial organic phase. The extracted lipids were dried overnight in a SpeedVac vacuum concentrator. Dried lipid extracts were reconstituted in 100 μl n-butanol/methanol 1:1 (v/v) and transferred into autosampler vials for analysis by LC-MS/MS.

LC-MS/MS sample acquisition was performed by injecting 5 µL of sample on an Ultimate 3000 UPLC coupled to a Q-Exactive Plus Orbitrap Mass Spectrometer (Thermo Scientific). Lipid separation was achieved using an Accucore C30 column (Thermo Scientific, 2.6 µm, 150×2.1 mm) maintained at 40 C and a flow rate of 300 µL/min. Mobile phase A (MPA) consisted of 60:40:0.1 (v/v/v) acetonitrile:water:formic acid + 10 mM ammonium formate. Mobile phase B (MPB) consisted of 10:90:0.1 (v/v/v) acetonitrile:isopropanol:formic acid + 10 mM ammonium formate. Initial mobile phase composition was 30% MPB, which was increased to 43% by 5 min, 50% by 5.1 min, 70% by 14 min, 99% by 21 min, then held at 99% MPB until 28 min, after which the column was returned to initial mobile phase conditions and allowed to re-equilibrate until 33 min. The mass spectrometer was operated with electrospray ionization in dual positive and negative mode. Capillary temperature was set to 200 C, sheath gas and aux gas flow rates were 60 and 20 respectively, and S-lense RF level set to 45. Data was acquired in both full scan MS and dd-MS/MS modes with resolution set to 70,000 and 35,000 respectively. Scan range was set from 400–1200 m/z in positive mode and 250–1500 m/z in negative mode. For MSMS acquisition, the collision energy was set to 28 eV. AGC target was set to 1e6 over 200 ms for full scan acquisition and 1e5 over 200 ms for dd-MS/MS acquisition. For MS/MS acquisition the isolation window was set to 1.0 m/z.

Acquired data was processed with Lipostar software (Version 2.1.0, Molecular Discovery) for peak picking, smoothing and retention time alignment. Background features were filtered using blank samples. Lipid identification was performed by querying the Lipid Maps structure database (LMSD) and using accurate mass database matches as well as MS/MS fragmentation patterns. The integrated peak areas of lipid features were normalized to total ion signal from each sample to account for variable material input and further normalized to corresponding lipid class internal standards. PCA plots, volcano plots and lipid set enrichment analysis were conducted in Rstudio using the lipidr package [80, 81] (version 2.15.1). Z-scores were calculated form the normalized peak areas to generate all heatmaps shown. LION lipid ontology enrichment analysis was performed using LION/web [82, 83].

### Quantitative RT-PCR

RNA was extracted from NHEK or HSE culture using RNeasy kit (Qiagen, 74104). 50 ng of RNA was used for PCR reaction with TaqMan RNA-to-C_T_ 1 step kit (Applied Biosystems, 4392938). TaqMan assay primers were purchased from ThermoFisher, and the assay number of each primer was listed in the Table S3. PCR reactions were performed using QuantStudio 7 Flex (Applied Biosystems) and relative quantification was measured by the calculation of $\triangle \triangle$CT.

### DiseaseLand data analysis

The expression of NLRP10 in skin inflammatory diseases was analyzed with QIAGEN OmicSoft Studio software (v12.7) using DiseaseLand Data (HumanDisease_B38_GC33_2024R1 release). Studies where NLRP10 expression was significantly different between lesional sites and normal control skin with an adjusted *p*-value of < 0.05 were selected. Dot plot summarizing the differential expression of NLRP10 across disease datasets was created in R.

### Statistical analysis

The data are shown as mean $\pm$ S.D. from more than three independent experiments including technical and biological replicates. The p values from pairwise comparisons were calculated using two-tailed Student’s *t*-test in GraphPad Prism (version 10.2.0).

## Supplementary information

Supplementary information

uncropped western blot

## Data Availability

The raw and processed RNA-seq data from this study were deposited in the GEO database with accession NO. GSE264616.
