# Supplementary material for: NLRP10 maintains epidermal homeostasis by promoting keratinocyte survival and P63-dependent differentiation and barrier function
Source: Cell Death Dis. 2024 Oct 18;15(10):759. doi: 10.1038/s41419-024-07146-y (PMC11492288; doi:10.1038/s41419-024-07146-y)
Supplement: Supplementary file 1 — Supplementary information [file 41419_2024_7146_MOESM1_ESM.docx]

**Table S1. gRNA sequences for NLRP10.**

| NLRP10 gRNA | Sequence |
| --- | --- |
| gRNA1 | CAAGGCCAGAAAGCCCCGGG |
| gRNA2 | CGGGATATGACCCTGTCTGA |

**Table S2. Antibodies used in this study.**

| Antibody | Vendor | Cat. Number | Dilution |
| --- | --- | --- | --- |
| Anti-NLRP10 | Millipore Sigma | MABC293 | 1:1000 |
| Anti-Caspase 8 | BD Biosciences | 551242 | 1:1000 |
| Anti-Cleaved Caspase 8 | Cell Signaling Technology | 9496 | 1:1000 |
| Anti-Cleaved Caspase 3 | Cell Signaling Technology | 9664 | 1:1000 |
| Anti-Cleaved Gasdermin D | Cell Signaling Technology | 36425 | 1:1000 |
| Anti-Cleaved Caspase 1 (p10) | Invitrogen | PA5-105049 | 1:1000 |
| Anti-GAPDH | Cell Signaling Technology | 5174 | 1:1000 |
| Anti-FLAG | ThermoFisher | MA1-91878 | 1:1000 |
| Anti-HA | Sigma | H6908-2ML | 1:1000 |
| Anti-Keratin 14 | Abcam | ab119695 | 1:500 |
| Anti-Keratin 10 | Abcam | ab9026 | 1:500 |
| Anti- β-catenin | Abcam | ab32572 | 1:250 |
| Anti-Filaggrin | LS Bio | LS-B13715 | 1:1000 |
| Anti-Caspase 14 | Cell Signaling Technology | 8519 | 1:1000 |
| Anti-Streptavidin | Invitrogen | S32356 | 1:1000 |
| Anti-p63α | Cell Signaling Technology | 4892 | 1:1000 |
| Anti-IKKα | Cell Signaling Technology | 2682 | 1:1000 |
| Anti-β-actin | Cell Signaling Technology | 3700 | 1:1000 |
| Goat-anti-Rabbit IgG, HRP | Invitrogen | 31460 | 1:10,000 |
| Goat-anti-Mouse IgG, HRP | Invitrogen | 62-6520 | 1:10,000 |
| Goat-anti-Rat IgG, HRP | Invitrogen | 31470 | 1:10,000 |
| Goat anti-Rabbit IgG, Alexa Fluor 488 | Invitrogen | A32731 | 1:1000 |
| Goat anti-Mouse IgG, Alexa Fluor 594 | Invitrogen | A11005 | 1:1000 |

**Table S3. TaqMan probes used in this study.**

| Gene | TaqMan Assay ID |
| --- | --- |
| Filaggrin | Hs00856927_g1 |
| Loricrin | Hs01894962_s1 |
| Claudin 4 | Hs00976831_s1 |
| Keratin 10 | Hs00166289_m1 |
| CHAC1 | Hs00225520_m1 |
| SCT2 | Hs01063215_m1 |
| Keratin 1 | Hs00196158_m1 |
| DSG1 | Hs00245189_m1 |
| RPL19 | Hs02338565_gH |


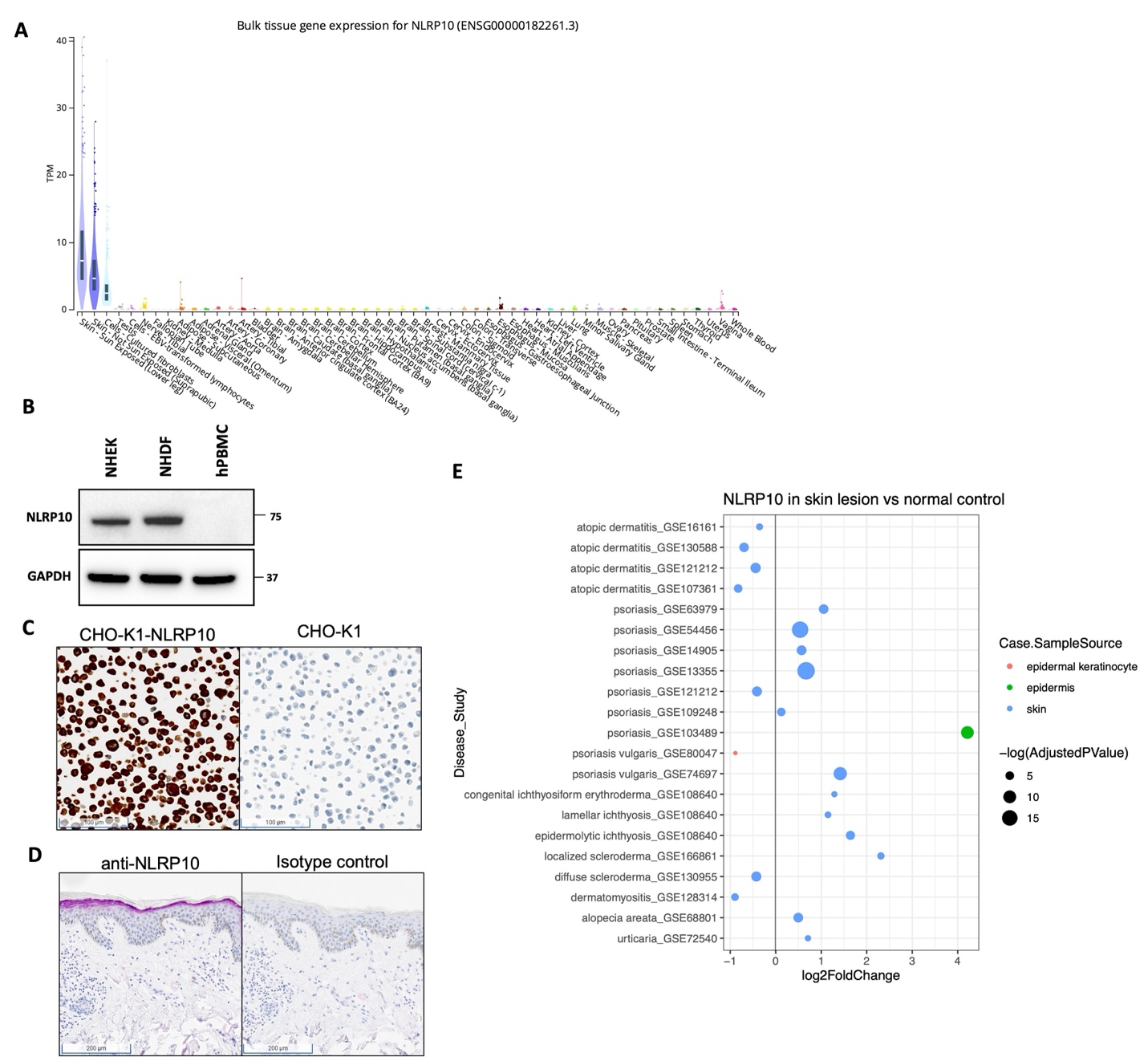


**Supplementary Figure 1. NLRP10 is specifically expressed in skin in humans.**

**A**, Genotype-Tissue Expression (GTEx) data showing human tissue specific expression of NLRP10. **B**, Protein expression of NLRP10 was analyzed by Western blotting in Normal human epidermal keratinocytes (NHEK), normal human dermal fibroblasts (NHDF) and human peripheral blood mononuclear cells (hPBMCs). GAPDH was used as a loading control. **C**, Representative immunohistochemistry (IHC) images of CHO-K1-NLRP10+ transfected cells and parental CHO-K1 cells stained with NLRP10. Scale bar: 100 µm. **D**, Representative IHC images of normal human skin sample stained with NLRP10 in purple and rat IgGa,k isotype control. Scale bar: 200 µm. **E**, Dot plot summarizing the differential expression of NLRP10 across various skin inflammatory diseases from the DiseaseLand datasets (HumanDisease_B38_GC33_2024R1 release). The size of the dot represents the negative log-transformed adjusted p-value, the position along the x-axis indicates the log-transformed fold change, and the color represents the sample source.


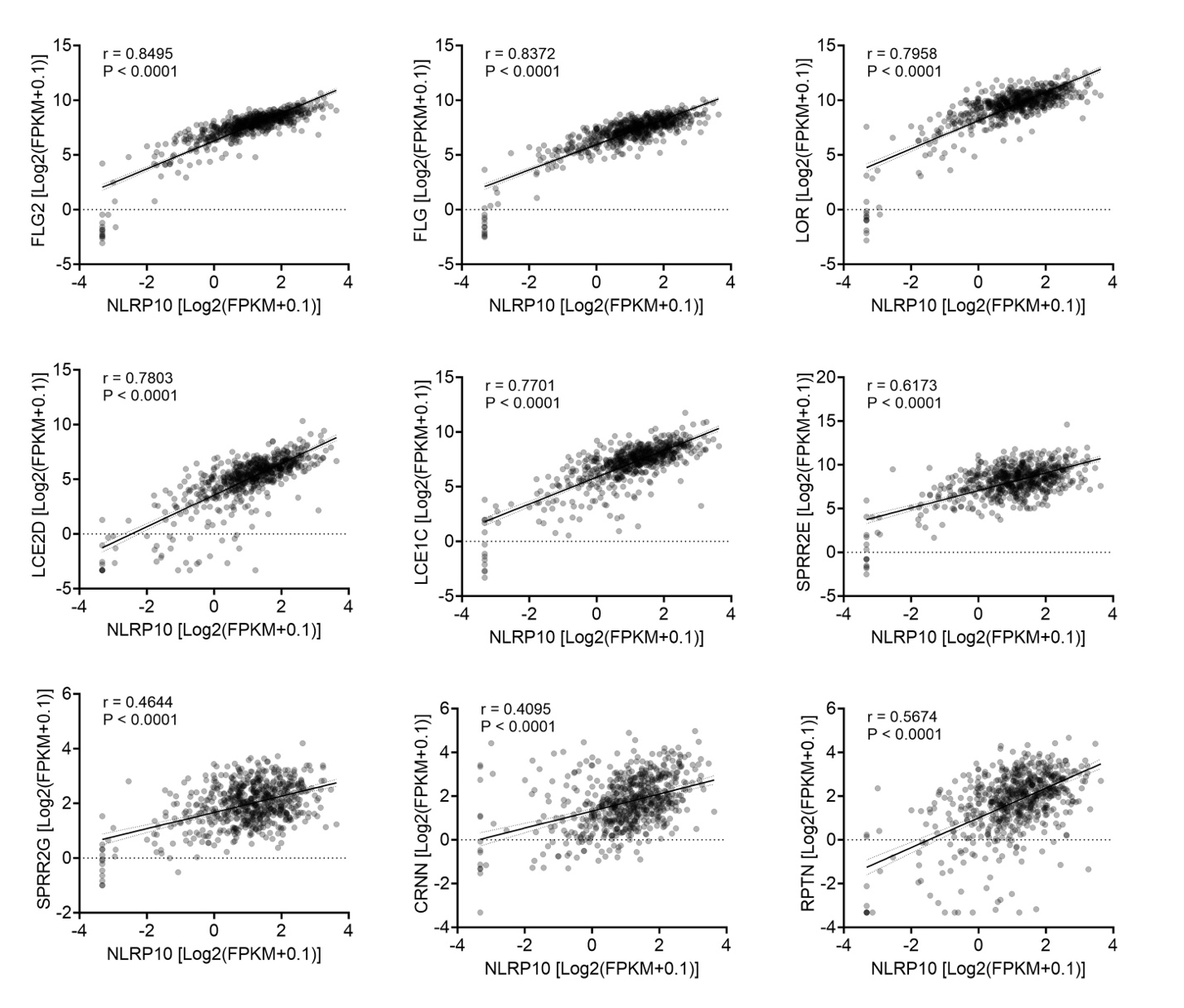


**Supplementary Figure 2. Genes involved in epidermal differentiation complex (EDC) are associated with NLRP10 expression in human skin.**

Scatterplot comparison of epidermal differentiation complex (EDC) genes with NLPR10 expressed as log2 (Fragments Per Kilobase of transcript per Million mapped reads+0.1). Pearson correlation coefficient r and P value are shown in the plot.


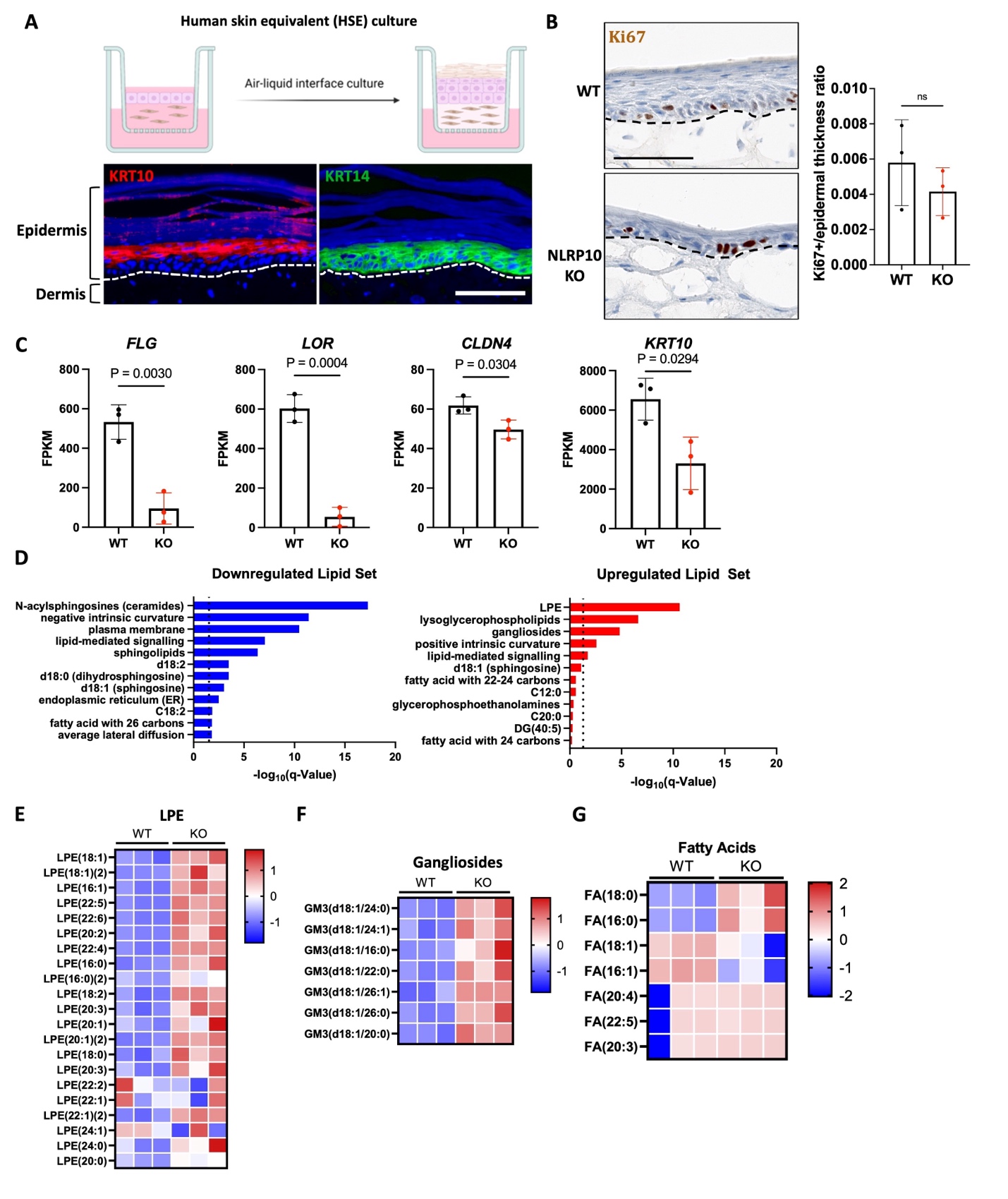


**Supplementary Figure 3. Lipid changes in NLRP10 KO HSEs.**

**A**, Schematics of human skin equivalent (HSE) culture construction, and representative images of HSEs with immunofluorescence staining of keratin 10 (KRT10) and keratin 14 (KRT14) showing different layers of epidermal differentiation. Scale bar: 20 µm. **B**, Representative images of Ki67 staining in WT and NLRP10 KO HSEs. The ratio of Ki67+ cells and epidermal thickness was quantified to account for developmental differences between WT and NLRP10 KO HSEs (n=3). Dashed lines highlight the boundary between the epidermis and the dermis within the HSEs. Scale bar: 100µm. **C**, Transcript levels of *FLG*, *LOR*, *CLDN4*, *KRT10* in WT or NLRP10 KO HSEs (n=3). FPKM: Fragments Per Kilobase per Million mapped reads (FPKM). **D**, Bar plots of lipid ontology enrichment analysis (LION) showing down-regulated lipid sets and up-regulated lipid sets. Dashed lines indicate q-value less than 0.05. **E-G**, Heatmaps from the NLRP10 KO and WT HSE lipidomics analysis showing upregulation of lysophosphatidylethanolamines (LPE) (**E**), gangliosides (**F**) and fatty acids (**G**) in NLRP10 KO HSEs (n=3). Heatmaps are colored by z-scores for each lipid species.


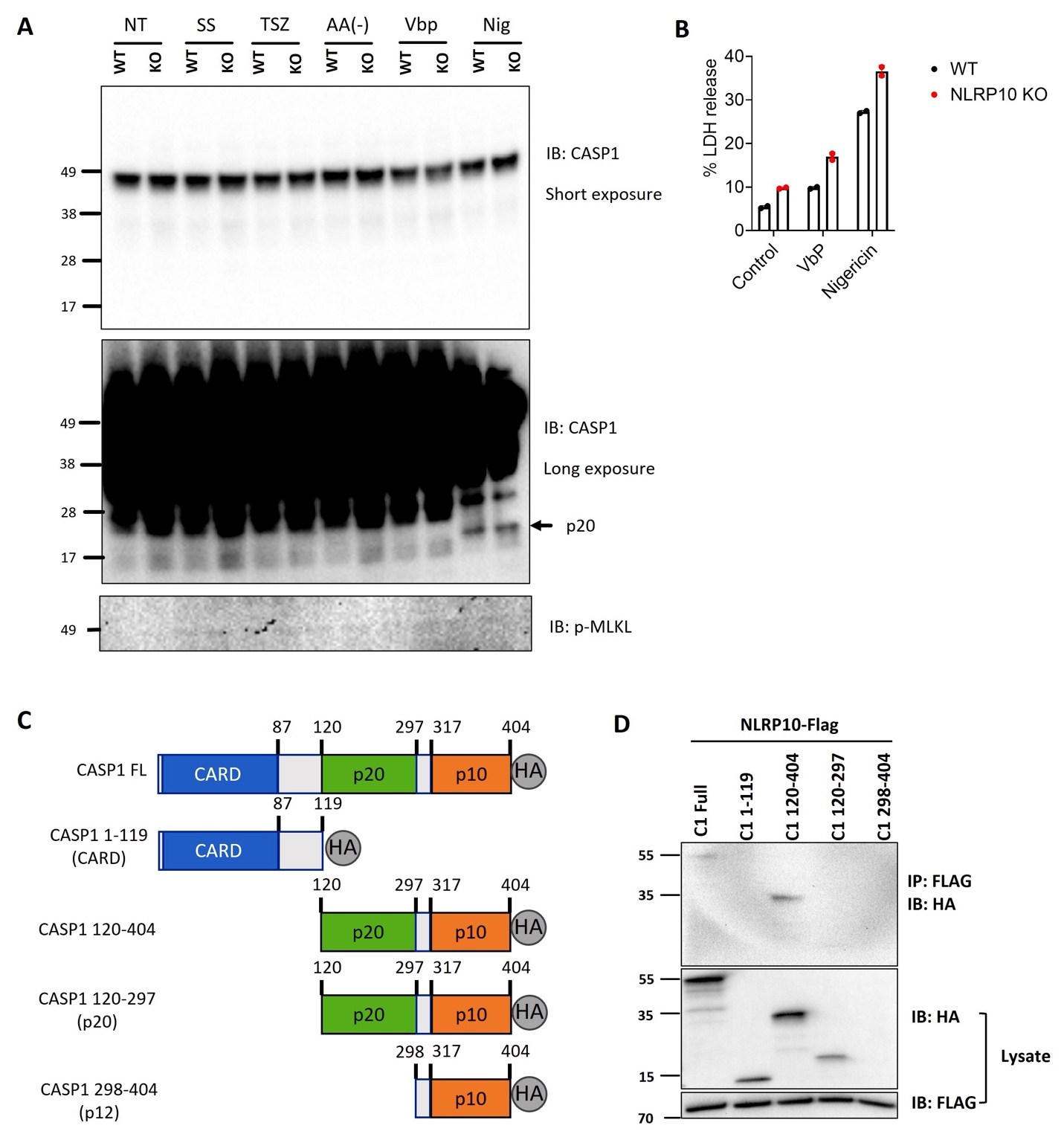


**Supplementary Figure 4. NLRP10 inhibits pyroptosis independent of caspase-1.**

**A**, Cell death pathways were analyzed by Western blotting. WT and NLRP10 KO NHEKs were treated similarly as in Figure 2. NT: non-treated; SS: staurosporine (1 µM, 4 h); TSZ: TNFα (20 ng/ml, 7 h)/SM-164 (100 µM, 7 h)/zVAD (20 µM, 7.5 h); AA(-): amino acids deprivation (1h); VbP (2 µM, 4 h); Nig: nigericin (6.7 µM, 4 h). Caspase 1 cleavage and phosphorylation of MLKL were analyzed for pyroptosis and necroptosis respectively. Long exposure of caspase-1 was used to reveal cleaved caspase-1 p20 fragment. **B**, LDH release from WT and NLRP10 KO NHEK treated with 2 µM VbP or 6.7 µM nigericin for 24 hours (n=2). **C**, Construct design of caspase 1 plasmids with different domains. **D**, Constructs in (**C**) were co-immunoprecipitated with NLRP10-FLAG and analyzed by Western blotting.


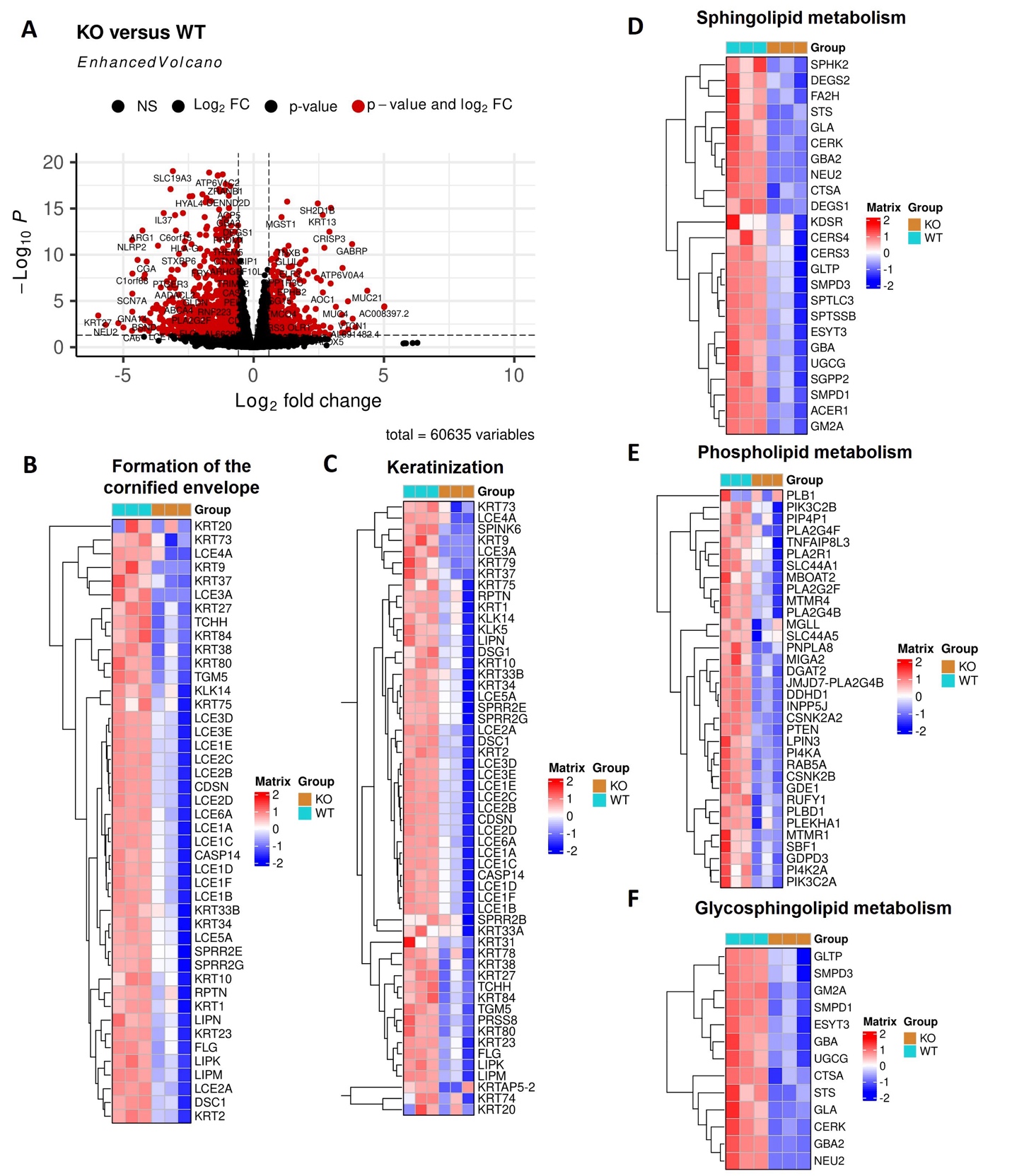


**Supplementary Figure 5. Gene expression changes in NLRP10 KO HSEs.**

**A**, Volcano plot highlighting differentially expressed genes (labelled in red, with adjusted P value < 0.05, fold change more than 1.5 fold or less than 2/3 fold) identified in NLPR10 KO HSEs compared to WT HSE. **B**-**F**, heatmaps of gene sets involved in keratinocyte terminal differentiation and lipid metabolism (n = 3): **B**, Formation of the cornified envelope; **C**, Keratinization; **D**, Sphingolipid metabolism; **E**, Phospholipid metabolism; **F**, Glycosphingolipid metabolism.


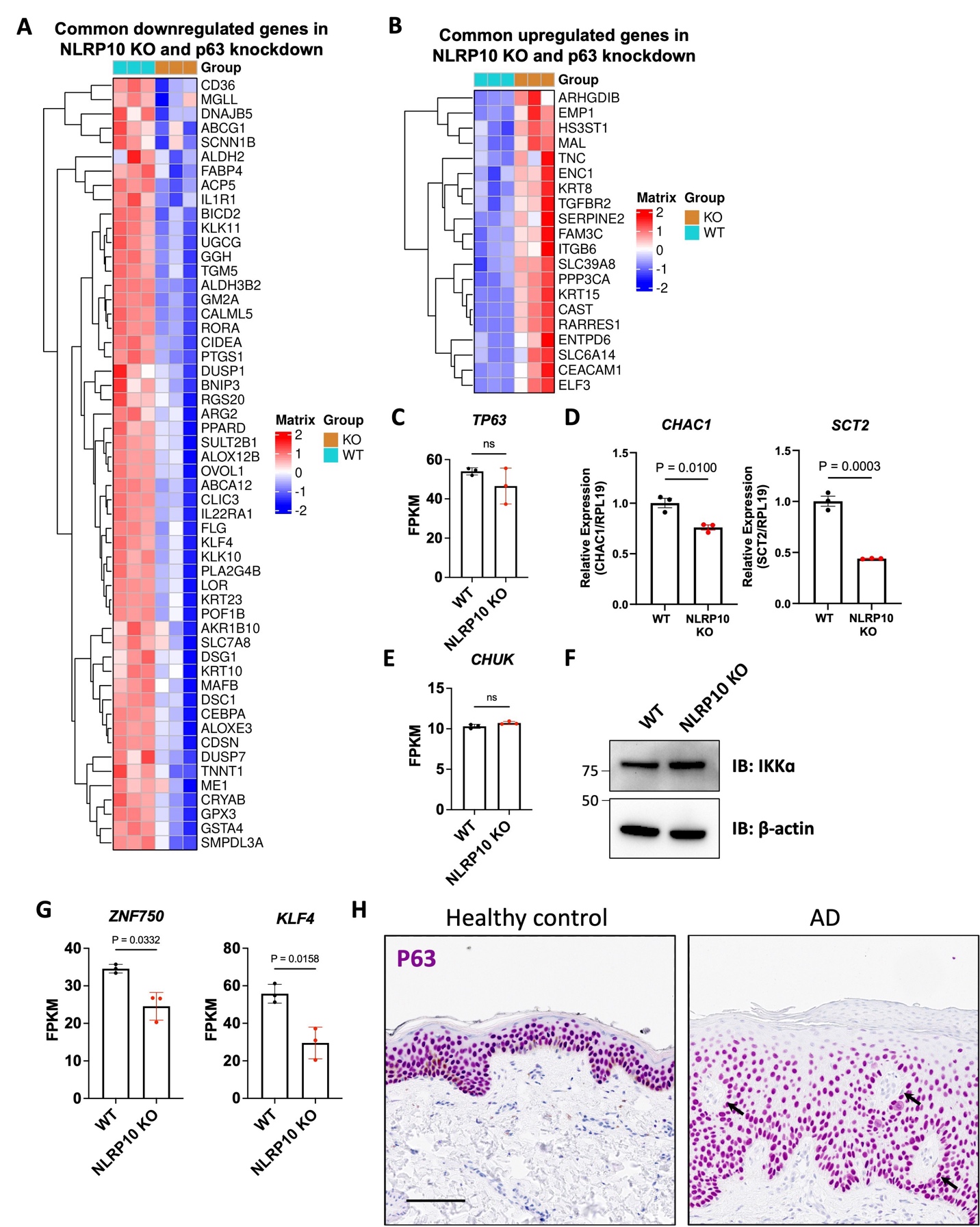


**Supplementary Figure 6. P63 signaling is downregulated in NLRP10 KO HSEs.**

**A**, Heatmap of commonly downregulated genes in NLRP10 KO and p63 knockdown HSEs (n = 3). **B**, Heatmap of commonly upregulated genes in NLRP10 KO and p63 knockdown HSEs (n = 3). **C**, *TP63* transcript levels in WT and NLRP10 KO HSEs (n=3). FPKM: Fragments Per Kilobase per Million mapped reads (FPKM). **D**, Relative expression of p63 target genes *CHAC1* and *SCT2* in WT and NLRP10 KO NHEKs (n=3). **E**, *CHUK* (gene encodes IKKɑ) transcript levels in WT and NLRP10 KO HSEs (n=3). **F**, Western blot of IKKɑ protein in WT and NLRP10 KO HSEs. **G**, *ZNF750* and *KLF4* transcript levels in WT and NLRP10 KO HSEs (n=3). **H**, Representative images of p63 staining in a healthy control skin sample and a severe atopic dermatitis (AD) sample. In healthy control and AD skin samples, there is strong p63 nuclear staining within the stratum basale and stratum spinosum. p63 expression is largely undetectable in the stratum granulosum and stratum corneum. Notably, in AD sample, cells within intraepithelial vesicles in the spongiotic foci do not stain for p63, as shown by arrows. Scale bars: 100µm.
