# Supplementary material for: NLRP10 maintains epidermal homeostasis by promoting keratinocyte survival and P63-dependent differentiation and barrier function
Source: Cell Death Dis. 2024 Oct 18;15(10):759. doi: 10.1038/s41419-024-07146-y (PMC11492288; doi:10.1038/s41419-024-07146-y)
Supplement: Supplementary file 2 — uncropped western blot [file 41419_2024_7146_MOESM2_ESM.pdf]

FLG

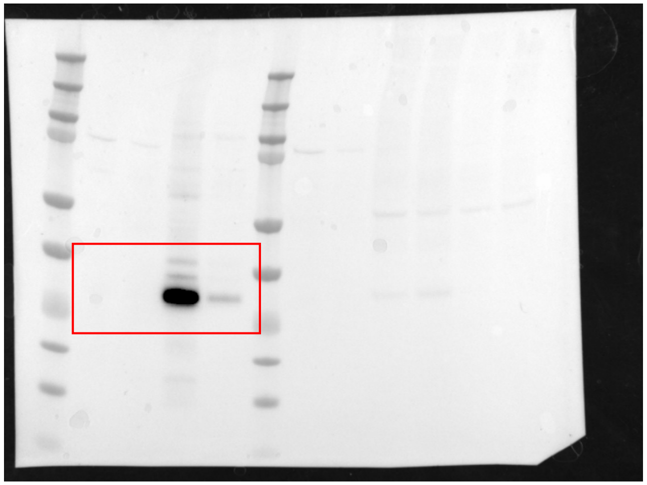

GAPDH

Casp14

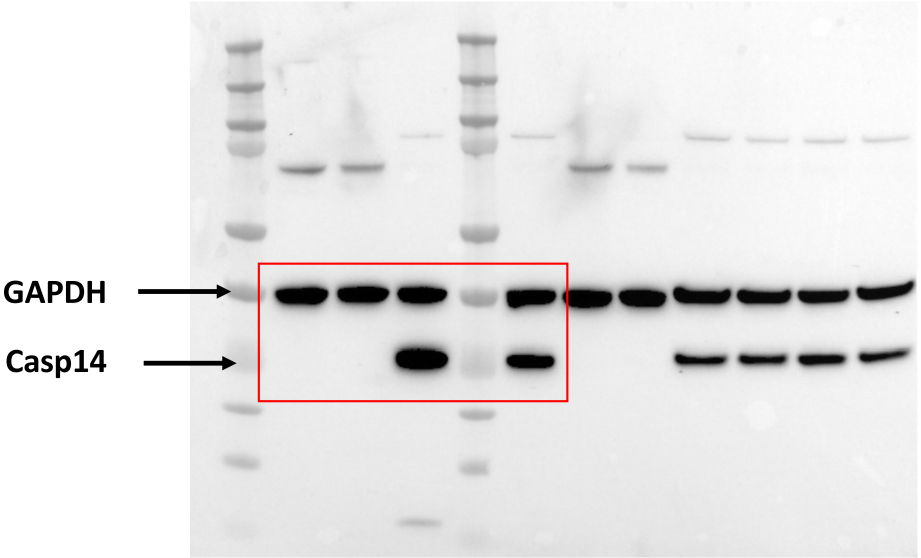

Uncropped immunoblot images of Fig. 2G

NLRP10

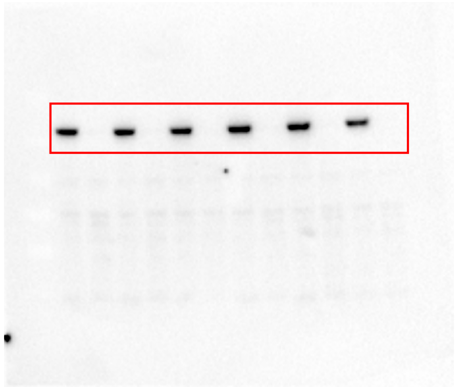

Clv. Casp8

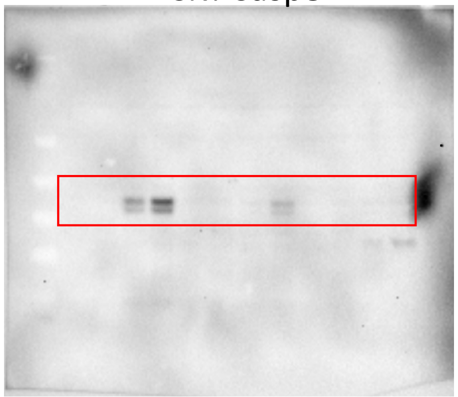

Clv. Casp3

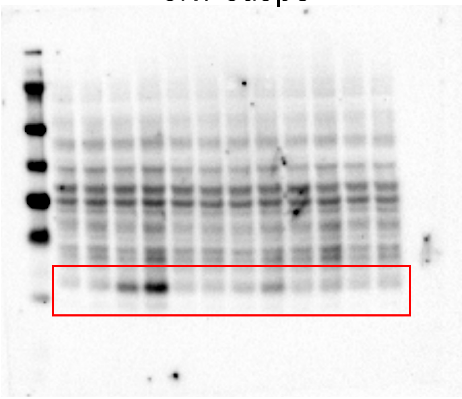

Clv. GSDMD

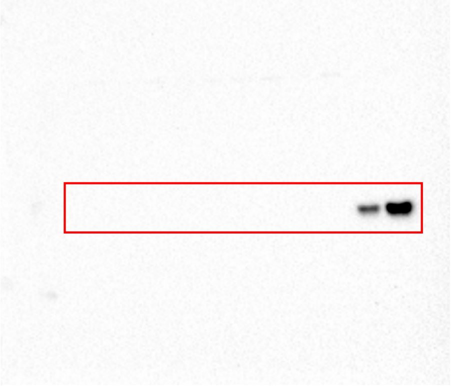

GAPDH

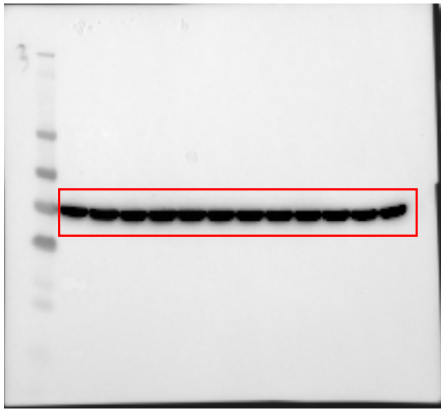

Uncropped immunoblot images of Fig. 4B

Clv. casp8

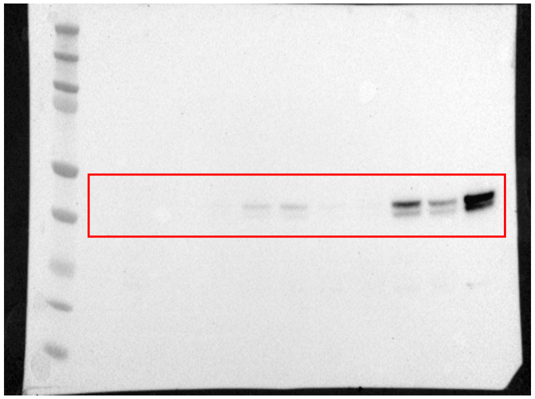

Clv. casp3

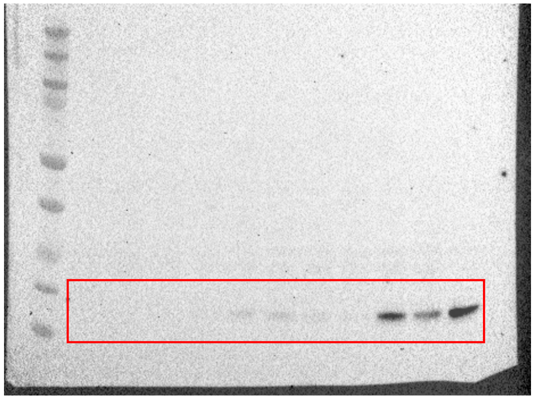

GAPDH

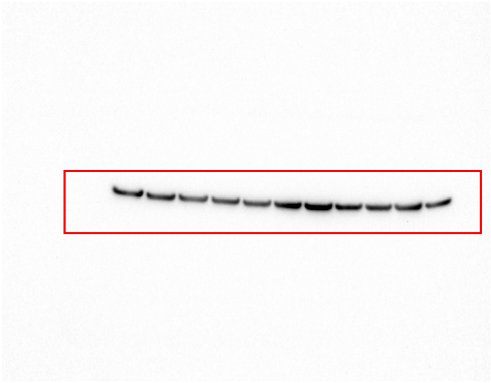

Uncropped immunoblot images of Fig. 4C

Clv. casp8

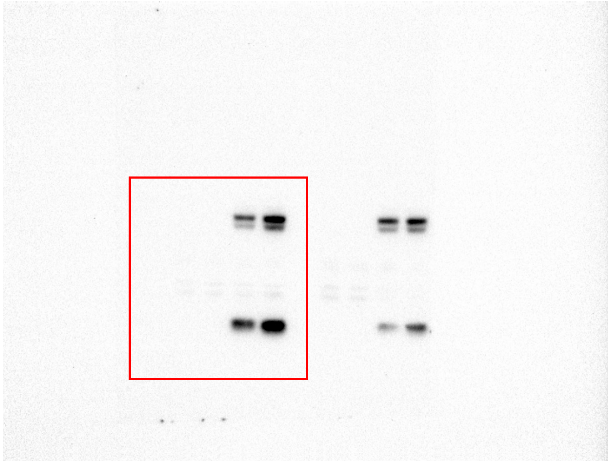

GAPDH

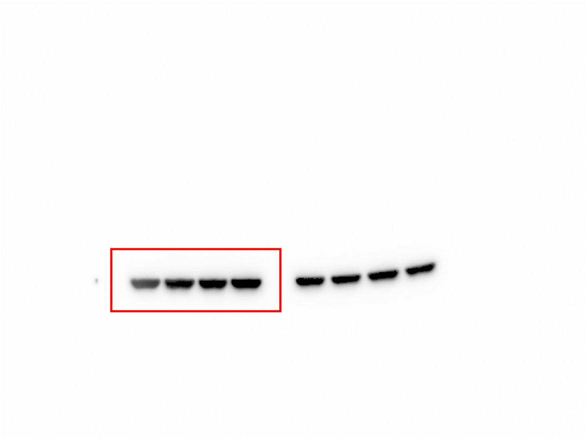

Uncropped immunoblot images of Fig. 4D

IP FLAG
IB NLRP10

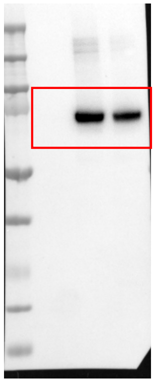

IP FLAG
IB HA

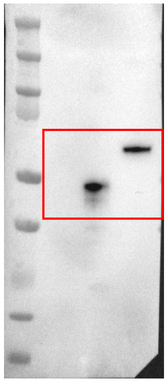

Lysate
IB NLRP10

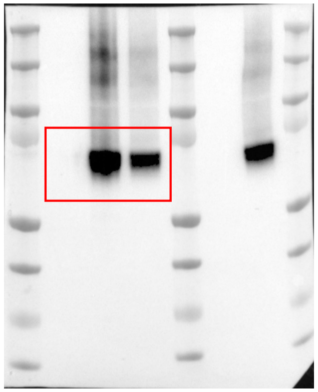

Lysate
IB HA

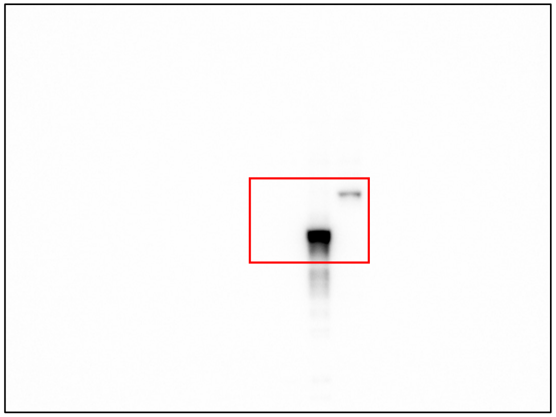

Lysate
IB GAPDH

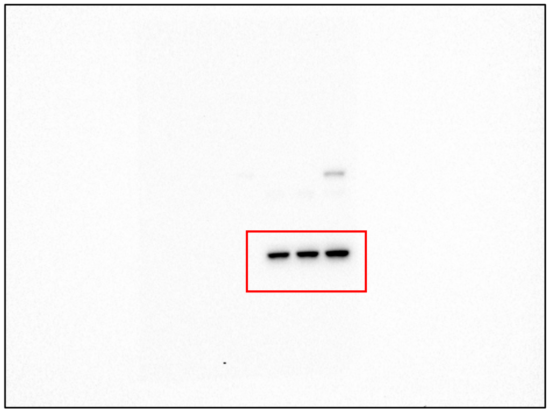

Uncropped immunoblot images of Fig. 5A

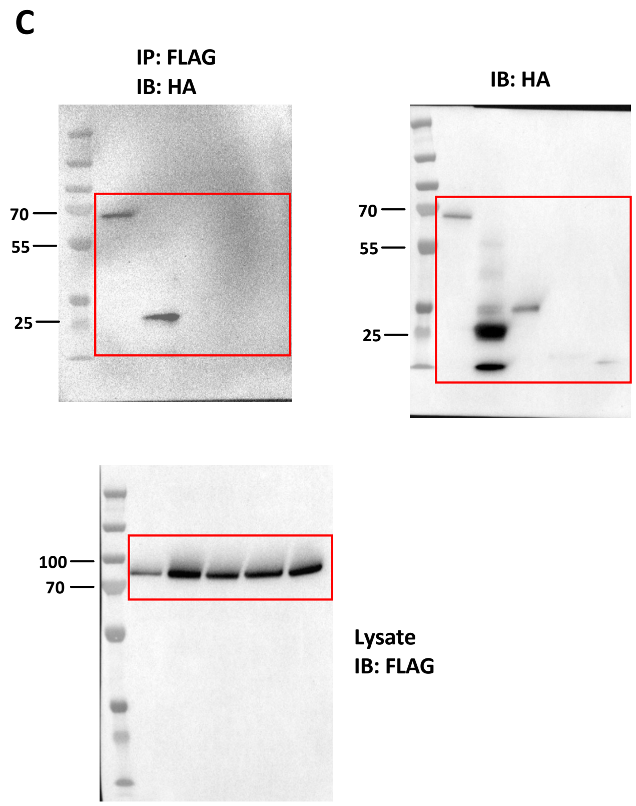

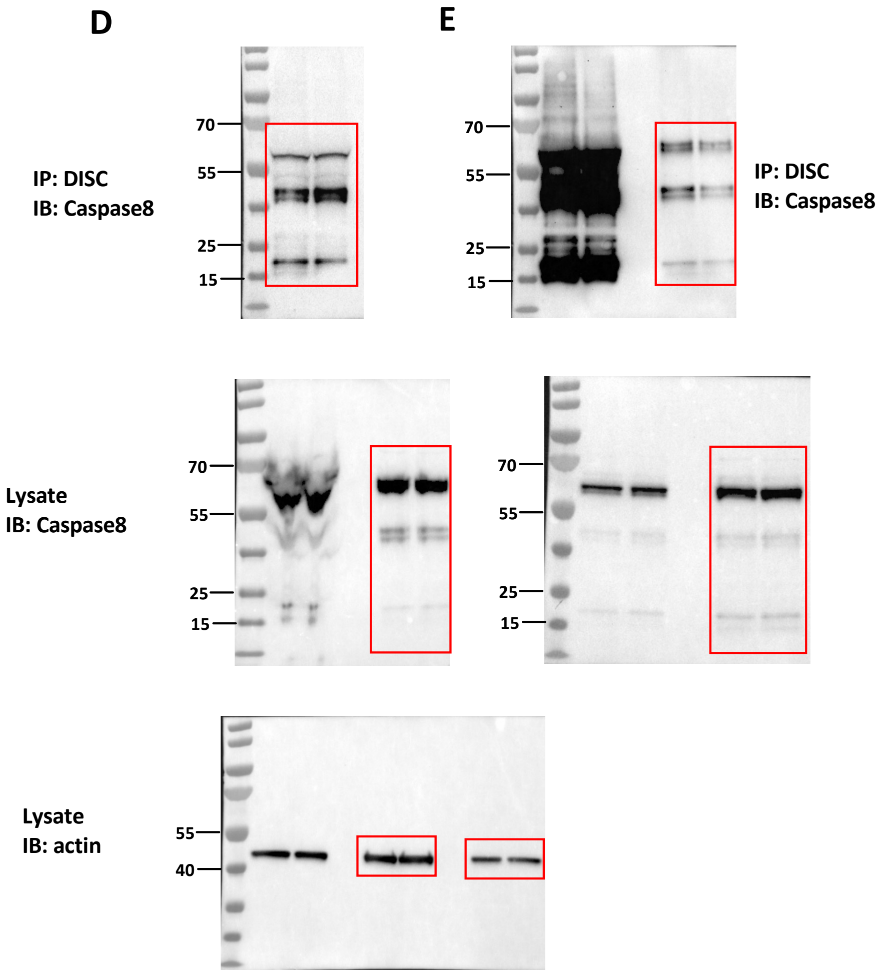

Uncropped immunoblot images of Fig. 5C-E

p63a

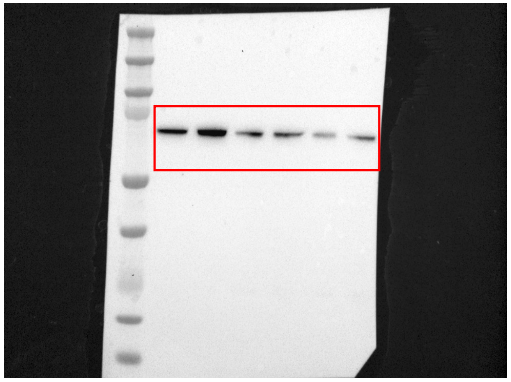

GAPDH

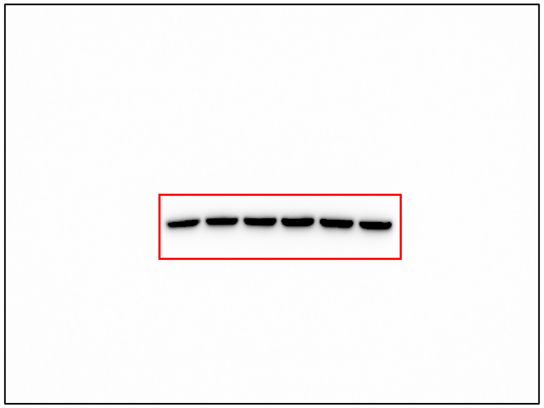

Uncropped immunoblot images of Fig. 6F

IP FLAG
IB NLRP10

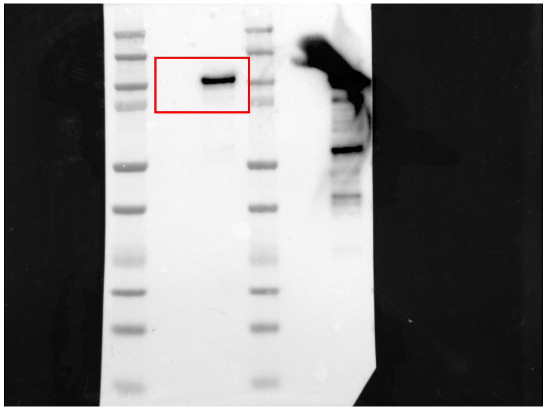

Lysate
IB NLRP10

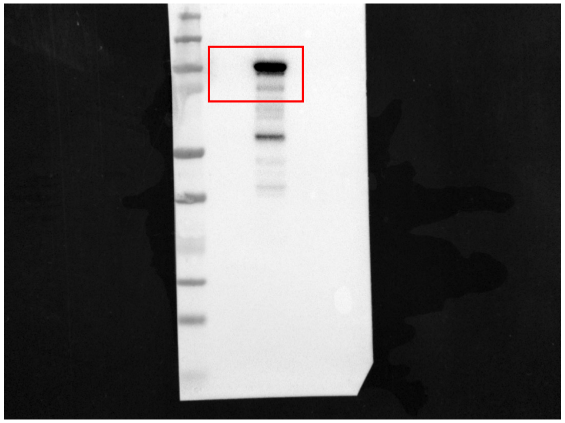

Lysate
IB GAPDH

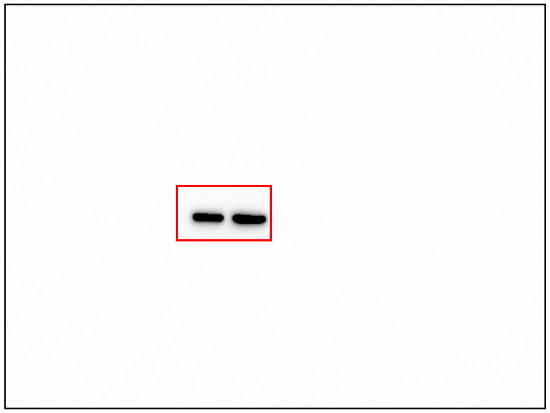

Uncropped immunoblot images of Fig. 7A

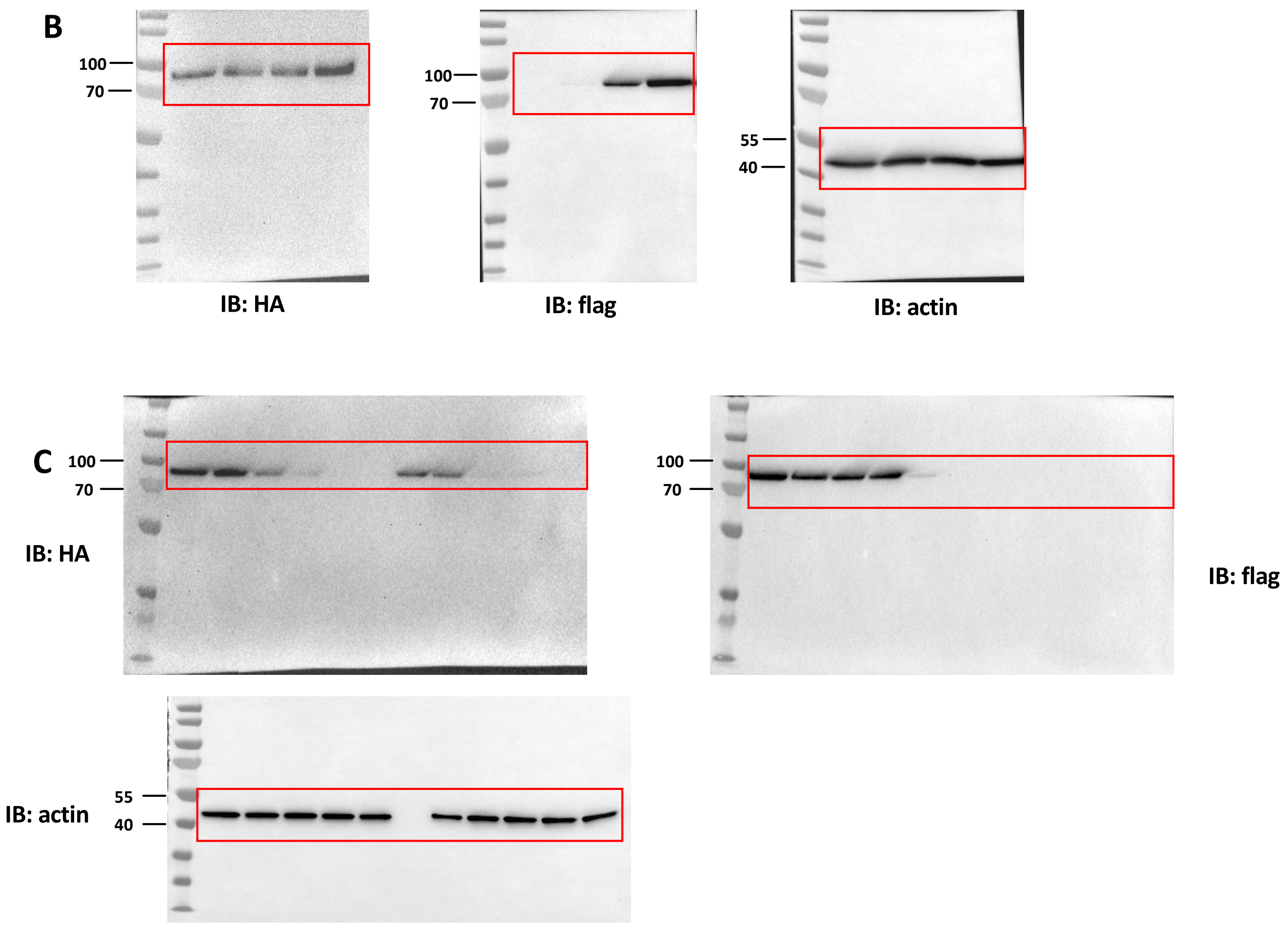

Uncropped immunoblot images of Fig. 7B-C

NLRP10

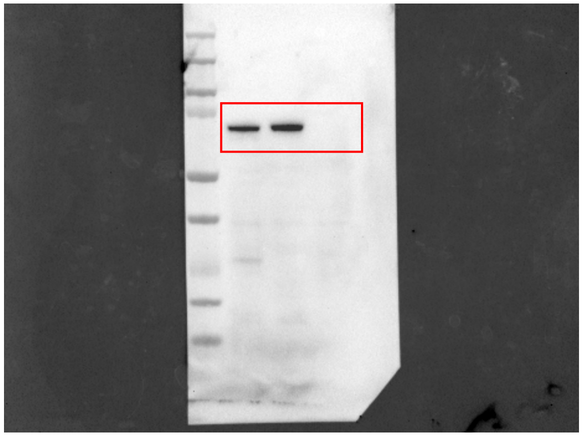

GAPDH

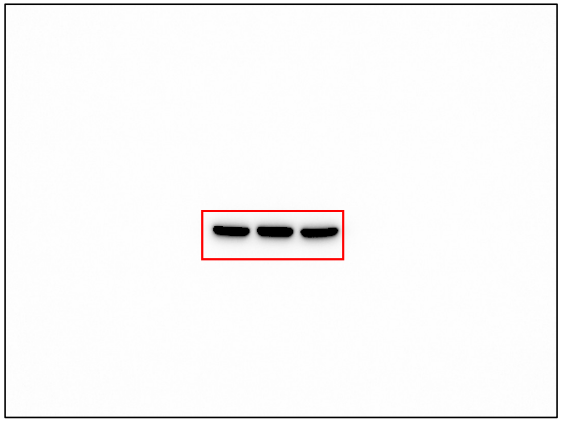

Uncropped immunoblot images of Supplementary Fig 1B

A

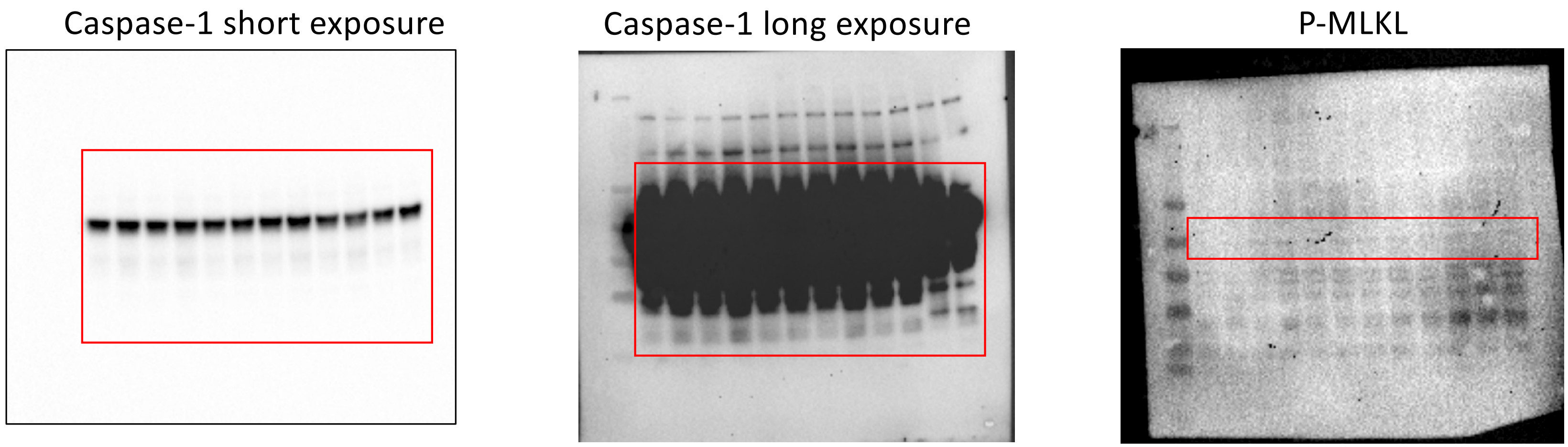

D

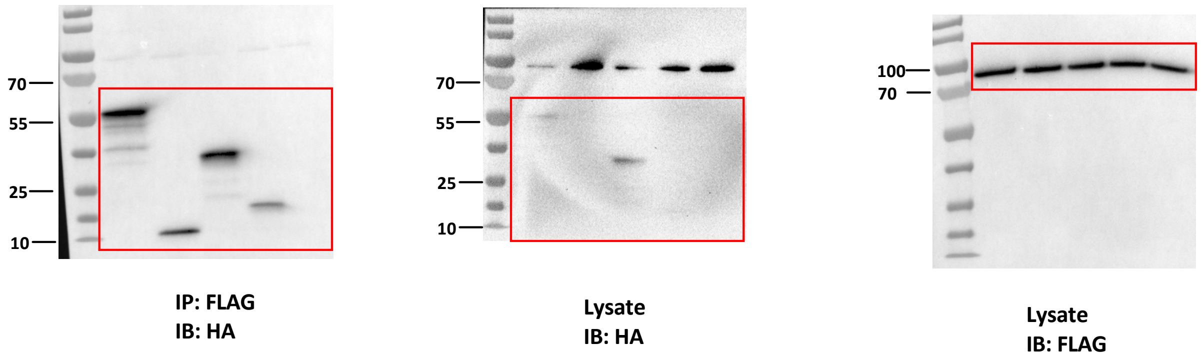

Uncropped immunoblot images of Supplementary Fig 4

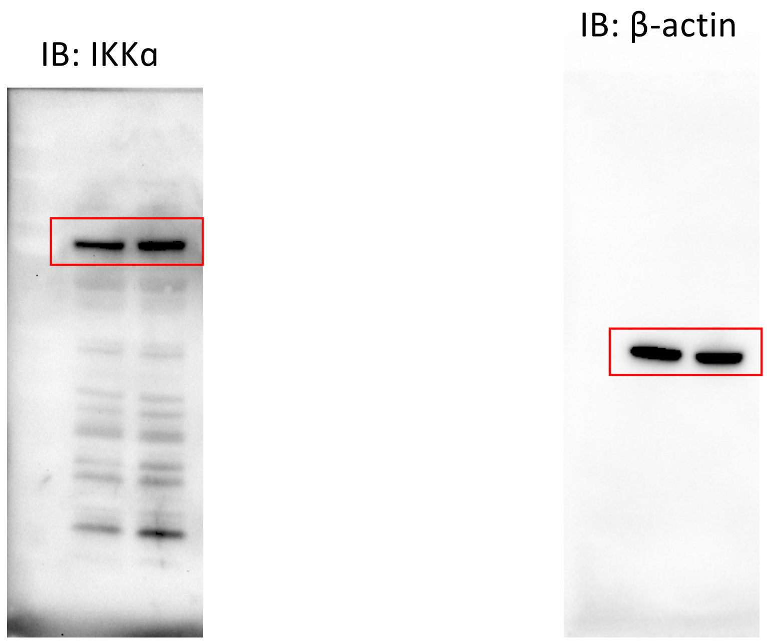

Uncropped immunoblot images of Supplementary Fig 6F
